# Monomer Structure and Discharge Power Influence the Multiscale Architecture of Plasma-Polymerized Hydrocarbon Nanoparticles

**DOI:** 10.1021/acspolymersau.6c00072

**Published:** 2026-06-16

**Authors:** Andrei Choukourov, Marco Tosca, Ján Šomvársky, Daniil Nikitin, Mariia Protsak, Iqra Wahid, Jan Hanuš, Veronika Červenková, Ronaldo Katuta, Krzysztof Tadyszak, Jiří Pánek, Eduardo Pitthan, Dmitrii Moldarev, Daniel Primetzhofer, Jakub Čížek, Eric Hirschmann, Maciej Oskar Liedke, Andreas Wagner, Maik Butterling, Hynek Biederman

**Affiliations:** † Faculty of Mathematics and Physics, Department of Macromolecular Physics, 138735 Charles University, V Holešovičkách 2, Prague 180 00, Czech Republic; ‡ ELI Beamlines Facility, The Extreme Light Infrastructure ERIC, Za Radnicí 835, Dolni Brezany 252 41, Czech Republic; § Institute of Macromolecular Chemistry CAS, Heyrovského nám. 2, Prague 162 06, Czech Republic; ∥ Department of Physics and Astronomy, Ångström Laboratory, 8097 Uppsala University, Box 516, Uppsala SE-751 20, Sweden; ⊥ Faculty of Mathematics and Physics, Department of Low Temperature Physics, Charles University, V Holešovičkách 2, Prague 180 00, Czech Republic; # Institute of Radiation Physics, 28414 Helmholtz-Zenrtrum Dresden-Rossendorf (HZDR), Bautzner Landstr. 400, Dresden 01328, Germany; ¶ Reactor Institute Delft, Department of Radiation Science and Technology, Faculty of Applied Sciences, Delft University of Technology, Mekelweg 15, Delft NL-2629 JB, The Netherlands

**Keywords:** plasma polymerization, hydrocarbon nanoparticles, radical concentration, free-volume size, cross-link density

## Abstract

Plasma polymer nanoparticles (pp-NPs) have emerged as linker-free nanocarriers for bioactive cargo and as nanofuels for laser-driven nuclear fusion, yet the structural factors governing their functionality remain poorly understood. Here, pp-NPs were synthesized via plasma polymerization of hexane, cyclohexane, and benzene to determine how monomer structure and specific energy input control radical concentration, hydrogen content, and hierarchical topology. Electron spin resonance and variable-energy positron annihilation lifetime spectroscopy establish that unquenched radical concentrations (10^14^–10^16^ spins mg^–1^) and free-volume size (0.5–0.6 nm) increase from aliphatic to aromatic monomers, whereas free-volume concentration decreases inversely. These trends arise from biradical formation in benzene plasma, steric constraints of aromatic rings, and denser packing caused by reduced hydrogen content, as confirmed by elastic recoil detection analysis. Higher discharge power unexpectedly reduces cross-link density in pp-NPs; statistical network reconstruction reveals that unsaturated carbon bonds reduce the concentration of elastically active network chains more strongly than dehydrogenation increases it. Atomic force microscopy demonstrates that aliphatic monomers yield smoother overall NP surfaces but with a more locally roughened profile (roughness exponent α = 0.68), whereas pp-benzene NPs exhibit higher global roughness yet smoother local morphology (α = 0.75). Upon deposition, pp-NPs assemble into porous coatings with interparticle voids tunable via NP size (65–560 nm). Together, these findings elucidate how plasma chemistry governs the hierarchical architecture of pp-NPs from subnanometer free volumes to nanoscale surface morphology and mesoscopic interparticle voids, establishing design principles for tailoring radical concentration, hydrogen content, and multiscale porosity for advanced applications.

## Graphical abstract

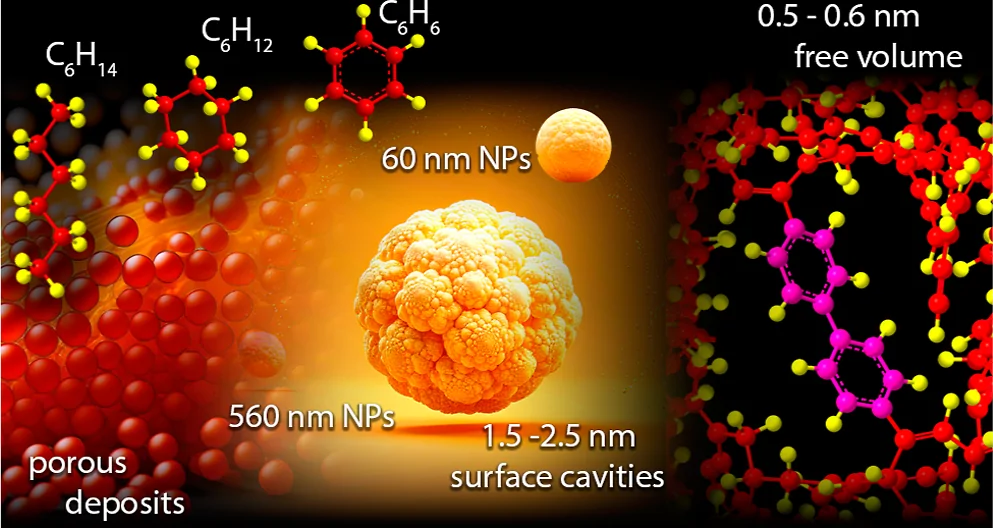

## Introduction

Experimental observations linking plasmas and polymers date back much earlier than the physical terms “plasma” and “polymer” were coined by Langmuir and Berzelius.^1,2^ Notably, as early as 1767, Lane reported spark discharges in various liquids, including aqueous suspensions of wheat flour, now known to contain starch-based polysaccharides, a class of natural polymers.^3^ Intentional studies of plasma-induced polymer formation began in the 19th century, when Berthelot, de Wilde, and Thenard described the condensation of organic solids from hydrocarbon vapors under electric discharges.^4,5^ Since then, and particularly from the mid-20th century onward, the field of these organic solids, known now as plasma polymers, has developed and diversified significantly.

Plasma polymers differ fundamentally from conventional polymers. They are best described as highly cross-linked hydrocarbon networks formed by electron impact-induced fragmentation of organic monomers in electrical discharges, followed by random polyrecombination of the resulting fragments. As a result, the chemical structure of plasma polymers bears little resemblance to the original monomers, and they lack the propagating sequences of regularly repeating units typical of conventional polymers. An exception occurs in plasma-assisted vapor phase deposition, where “evaporation” of polymer supplies relatively long oligomeric species for plasma polymerization.^6–9^ At relatively low pressures, where the mean free path is high and the collision frequency is low, organic fragments tend to recombine on surfaces adjacent to the plasma, forming thin films.^10^ The strongly nonequilibrium conditions of plasma polymerization preclude the use of conventional thermodynamic concepts to describe the mechanisms; instead, various semiempirical models have been developed.^4,5^ Thin films of plasma polymers are typically free from pinholes, adhere strongly to substrates, and exhibit tunable dielectric and optical properties, making them the subject of extensive research.^11–14^

The gas-phase formation of plasma polymer nanoparticles (pp-NPs) in hydrocarbon plasmas has also been reported. Historically, this phenomenon was often considered undesirable, particularly in microelectronics manufacturing, where NP accumulation on surfaces could impair the plasma processing quality. As a result, research on pp-NPs was long confined to fundamental studies, primarily driven by plasma physics and astrophysics communities interested in the properties of dusty (complex) plasmas, rather than the pp-NPs themselves.^15–18^ In recent decades, however, a shift toward sustainable materials and processes has renewed interest in pp-NPs across various fields, especially in materials science. From a sustainability perspective, pp-NPs offer significant advantages: their synthesis requires only small amounts of inexpensive and abundant precursors, proceeds without extreme temperatures and solvents, and generates minimal waste.^19^

Emerging applications have revealed the strong potential of pp-NPs, particularly as linker-free nanocarriers of bioactive cargo.^20–26^ Direct covalent attachment of antibodies, enzymes, RNAs, and drugs has been achieved by exploiting the free radicals inherent to pp matrices, thereby avoiding conventional wet chemistry protocols for linker attachment and multiple purification steps. These bioengineered pp-NPs have demonstrated efficient cellular uptake and the ability to deliver drugs intracellularly while maintaining biocompatibility even at higher concentrations. Furthermore, such biomodified pp-NPs can be used to decorate a wide range of biomaterial scaffolds with complex geometry, where other modification strategies fail.^27,28^

Another promising application involves the use of pp-NPs as nanofuels for laser-driven proton-boron nuclear fusion. This method offers an attractive alternative to traditional magnetic-confinement fusion schemes by initiating nuclear fusion between hydrogen and boron nuclei through irradiation of hydrogen- and boron-rich materials with high-energy laser pulses.^29^ In this case, the fusion reaction is neutronless, thereby avoiding challenges associated with neutron-induced radioactivity, and is envisioned for “green” electricity generation and other potential applications. To enhance laser energy absorption and proton acceleration, porous materials with high hydrogen content are needed, while environmental considerations require sustainable synthesis approaches, making porous deposits of pp-NPs attractive candidates.^30,31^

These applications highlight the importance of radical concentration, cross-link density, chemical composition (including hydrogen content), and surface morphology of the pp-NPs. However, detailed and comprehensive knowledge of these properties is limited. In this work, we address this knowledge gap by systematically exploring pp-NPs prepared from hexane, cyclohexane, and benzene, which are hydrocarbon monomers of similar molar masses but distinct chemical structures. These monomers represent a progression from a linear saturated molecule (hexane) to a saturated cyclic structure (cyclohexane) and finally to an aromatic system (benzene). This selection enables us to disentangle how backbone flexibility and aromaticity influence plasma-driven monomer fragmentation pathways and the resulting structural topology, hydrogen content, and radical concentration in pp-NPs. Our aim is to provide deeper insight into these interrelationships and suggest future research directions.

## Experimental Section

### Synthesis of pp-NPs

Plasma polymer NPs were prepared by igniting rf glow discharge (13.56 MHz) in mixtures of the vapors of hydrocarbon monomers and Ar (Linde, purity 99.99%). A gas aggregation cluster source (GAS) was adapted to perform plasma polymerization (Figure 1). The GAS consisted of a stainless-steel cylindrical reactor with an inner diameter of 10 cm and external water cooling. The top flange of the GAS was equipped with a stainless-steel electrode of 3 in. in diameter, whereas the bottom flange was equipped with an orifice of 3 mm in diameter. The entire GAS assembly was installed vertically on a vacuum chamber (not shown in Figure 1 for simplicity), where pp-NPs were collected on Si substrates. The system was pumped by rotary and diffusion pumps to a base pressure of 10^–3^ Pa in the deposition chamber. Vapors of monomers were introduced from glass flasks connected to the top flange of the GAS via needle valves. Three monomers were chosen based on close molar masses but different chemical motifs: hexane C_6_H_14_ (*M* = 86 g/mol, Sigma-Aldrich, CAS-No: 110-54-3, assay ≥99%), cyclohexane C_6_H_12_ (*M* = 84 g/mol, Sigma-Aldrich, CAS-No: 110-82-7, assay ≥99%), and benzene C_6_H_6_ (*M* = 78 g/mol, Sigma-Aldrich, CAS-No: 71-43-2, assay ≥99%).

**Figure 1:**
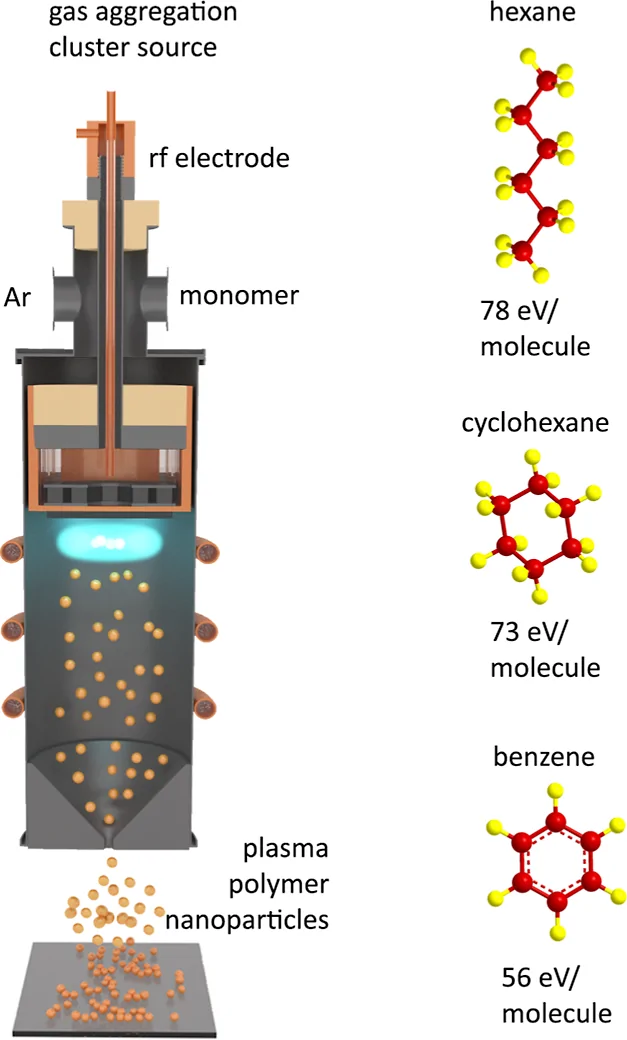
Scheme of the GAS used for plasma polymerization of three hydrocarbon monomers (adapted with permission from ref 32, Copyright 2021 Elsevier). The dissociation energies of all bonds in the monomer molecules (*E*_D_, eV/molecule) are shown according to refs 33 and 34 .

Plasma polymerization was performed at a total gas flow of 4.8 sccm, a total pressure of 50 Pa, and a monomer partial pressure of 3 Pa, resulting in a monomer concentration of 6 vol %. Rf power of 30 and 100 W was used. Considering close molar masses of the monomers, at a given rf power, plasma polymerization proceeded at approximately the same energetic conditions, which can be described either by the Yasuda parameter *W*/*MF* (where *W* is the discharge power, *M* is the molar mass of monomer, and *F* is the flow rate of monomer) or, even more generically, by the mean energy supplied per monomer molecule *E*_mean_ = *WV*_m_/*FN*_A_ (where *V*_m_ is the molar volume of an ideal gas and *N*_A_ is the Avogadro constant).^35,36^ For 30 and 100 W used, *E*_mean_^30W^ is 87 eV/molecule and *E*_mean_^100W^ is 290 eV/molecule; if compared to the sums of bond enthalpies of the monomers (Figure 1), these values point to a monomer-deficient regime with strong fragmentation of monomer molecules.

### Characterization of pp-NPs

The morphology of pp-NPs and their size distributions were analyzed by acquiring SEM images. A microscope (SEM JSM-7200F, Jeol Ltd., Tokyo, Japan) was equipped with a high-brightness Schottky field-emission (T-FE) electron gun, In-Lens Schottky Plus TM technology-based electron optics, and a hybrid objective lens.

AFM (Ntegra Prima, Nt-MDT) with supersharp tips (SHR75, BudgetSensor, spring constant 3 N/m, diamond-like carbon with guaranteed radius of 1 nm) was used to explore the surface of individual pp-NPs. Scanning was performed on submonolayers of pp-NPs in ambient air in an intermittent contact mode. Examples and details of the AFM protocol can be found in Figure S1 of the Supporting Information. First, groups of pp-NPs were identified at a lower magnification of 20 × 20 μm, and then this area was zoomed in to choose individual pp-NPs. The upper hemisphere of individual pp-NPs was scanned with a scan size of 200 × 200 nm and resolution of 512 × 512 data points. Under these conditions, the lateral resolution of the AFM scans is given by the tip radius of 1 nm. Raw 200 × 200 nm AFM scans were flattened by subtracting second-order polynomials and height–height correlation functions were obtained from the flattened images. Roughness exponent α was calculated from the slope of the linear regions of the height–height correlation functions plotted in log–log coordinates, as shown in Figure S2 of the Supporting Information.

The chemical composition of pp-NPs was studied by time-of-flight-elastic recoil detection analysis (ToF-ERDA), FTIR, and XPS. ToF-ERDA was performed using ^127^I^8+^ ions at 36 MeV energy. The ions were incident at 67.5° to the surface normal, while the recoil angle was 45°. Element depth profiles were obtained using the CONTES software. The acquisition time was set to 50 s to limit the sample degradation from the ion beam, see Figure S3 of the Supporting Information for details. To further verify the reliability of the data, hydrogen depth profiling using resonant nuclear reaction analysis (NRA) was performed, see Figure S4 of the Supporting Information for details.

The FTIR measurements (Bruker Equinox 55) were performed in a reflectance–absorbance mode on samples deposited on gold-mirror-covered silicon wafers, with a resolution of 4 cm^–1^ and 200 scans.

XPS spectra were acquired using a spectrometer (Phoibos 100, Specs) with an Al Kα X-ray source (1486.6 eV, 200 W, Specs). The wide and high-resolution spectra were recorded at a pass energy of 40 eV (dwell time 100 ms, step 0.5 eV, 1 scan) and 10 eV (dwell time 100 ms, step size 0.05 eV, with 10 repetitions), respectively. Quantitative analysis was performed using the CasaXPS software (v. 2.3.19PR1.0, Casa Software Ltd.), with a charge correction applied to the adventitious C 1s peak at 285.0 eV.

Radical concentration in pp-NPs was studied by electron spin resonance (ESR) spectroscopy. The measurements were conducted with a Bruker ELEXSYS 540 X-band spectrometer equipped with a 049X microwave bridge (Super X) and a Bruker ER4108 TMHS slim design resonator. Alanine was used as a primary spin concentration standard (BRUKER ER213 ASC Alanine Spin Concentration Sample E3005324, Ser-No.: 201812-10, 1.7 × 10^17^ spins). Using this primary standard, a secondary internal standard was calibrated (Al_2_O_3_:Cr^3+^ doped monocrystal, 4.76 × 10^15^ spins). All the measurements were started 1 h after sample preparation, and all samples were compared with the secondary standard signal. The following settings were applied for measurements: microwave power is 20.07 mW (10 dB); modulation amplitude is 1 G; frequency modulation is 100 kHz; the time constant is 5.12 ms; conversion time is 20.48 ms; gain is 60 dB; resolution is 2048 points; and room temperature is 294.15 K, 15 accumulations per spectrum. The *g*-factor was estimated at 2.004 relative to the DPPH free radical standard, assuming its *g*-factor is 2.0036. Because of a broad distribution of carbon-centered unpaired electrons in plasma polymers, no specific radical species could be uniquely assigned. The ESR spectra were therefore interpreted in terms of the overall spin density (i.e., total concentration of unpaired electrons) rather than individual radical identities. No external scavenger was employed in the ESR measurements, except for minor postsynthesis interactions with ambient species (e.g., oxygen), as discussed in the text.

Variable energy positron annihilation lifetime spectroscopy (VEPALS) was used to investigate free volumes in pp-NPs. The measurements were performed at HZDR, Dresden, Germany, using a pulsed monoenergetic positron beam, MePS,^37^ operated at the ELBE superconducting electron linear accelerator.^38^ The pulsed positron beam had a diameter of approximately 3 mm. The implantation energy of positrons (i.e., the kinetic energy of the positrons) was adjusted in the range of 0.5–11.0 keV to enable depth profiling. Consequently, the measured positron lifetime corresponds to the fraction of the film volume beneath the beam cross-section at the mean depth defined by the positron implantation energy. A digital spectrometer equipped with a CeBr_3_ scintillator coupled to a Hamamatsu R13089-100 photomultiplier was employed for the positron-lifetime measurements.^39^ Waveforms from the detector were sampled with a Teledyne SP Devices ADQ14DC-2X digitizer featuring a 14 bit vertical resolution and a 2 GS/s sampling rate. The spectra were fitted with a multiexponential function to obtain the positron lifetimes, following the procedure developed in our previous work.^40^ Five lifetime components (τ_1_–τ_5_) with corresponding intensities (*I*_1_–*I*_5_) were required for an adequate fit. Their potential assignments are discussed in the relevant section and also in Figures S5–S7 of the Supporting Information.

### Statistical Reconstruction of Plasma Polymer Network Structure

The theory of branching processes (TBP)^41–45^ was employed to assess the network density in pp-NPs expressed as the concentration of elastically active network chains (EANC, *n*_e_), a key descriptor of highly cross-linked polymer networks. Since in plasma polymerization, reaction pathways, rate constants, and even the set of reactive intermediates are generally unknown, the network should be reconstructed from incomplete structural information, that is, from data characterizing the end product rather than the formation process itself. TBP provides a natural framework for reconstructing network structures when only limited information about the final structure is available. Rather than modeling the underlying chemistry, the method generates random networks that are statistically consistent with the prescribed structural constraints. Missing information is replaced with a controlled assumption of randomness.

The pp-NP network was statistically reconstructed by random pairing of predefined *building units* (BUs) under constraints imposed by nearest-neighbor connectivity. BUs comprised carbon and hydrogen atoms and, in the case of benzene, benzene rings. Connectivity between BUs was described using a limited set of *oriented links* (OLs) representing single, double, and triple carbon–carbon bonds, as well as C–H bonds. The statistical distribution of these links was constrained by the experimentally measured H/C ratio. The molar fractions of BUs were encoded in a generating function, which served as the statistical input for TBP. Once the consistent set of parameters was found, TBP directly provided the concentration of EANC. Under normalization used, this quantity represents the number of EANC per carbon atom and serves as a measure of network density in pp-NPs. Details of all mathematical definitions and assumptions are provided further in the text, while computational details are provided in the Supporting Information.

## Results and Discussion

Plasma polymerization of hexane, cyclohexane, and benzene results in the formation of pp-NPs in the gas phase, while a differential pressure between the GAS and the deposition chamber transports them to substrates, where they can be collected. For example, Figure 2a shows AFM height images at different magnifications of a submonolayer of pp-benzene NPs prepared at 30 W and deposited on a Si substrate. The lower-magnification images show that the pp-NPs land randomly on the substrate, piling on one another and forming agglomerates rather than forming a densely packed monolayer. The higher magnification images highlight that the pp-NPs are spherical and have a developed surface. Flattening the high-resolution hemispherical image of a single NP reveals that its surface texture consists of nanoscale bumps and valleys, a structure previously termed a cauliflower.^20,46,47^

**Figure 2:**
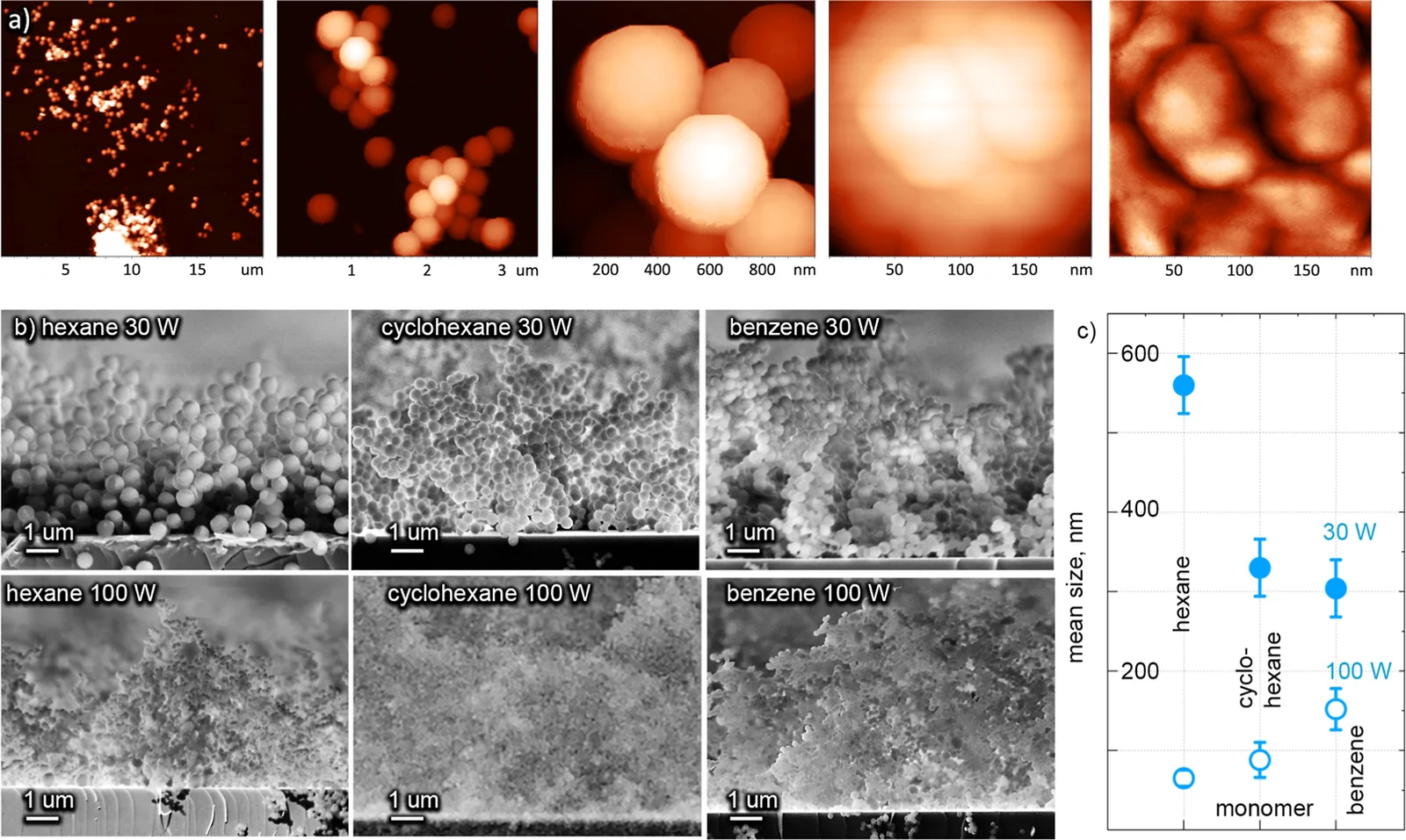
(a) AFM top-view images at different magnifications showing a submonolayer of pp-NPs prepared from benzene at 30 W; the last two panels present the raw image and the flattened top hemisphere of a single NP; (b) cross-sectional SEM images; and (c) mean sizes of pp-NPs prepared from different monomers at 30 and 100 W. For all measurements, pp-NPs were deposited on Si substrates.

Deposition of pp-NPs produces highly porous assemblies, as confirmed by SEM cross-sectional images of such coatings prepared from different monomers at 30 and 100 W discharge powers (Figure 2b). While avoiding definitive claims that the landing of pp-NPs occurs in a strictly ballistic regime, it can be reasonably stated that they deposit without substantial relaxation at the growth front, predominantly sticking to the first point of contact. This regime leads to a rough structure with significant lateral growth,^48^ which can be beneficial in fields where highly porous nanomaterials are of primary concern.

Complementary measurements of N_2_ adsorption/desorption isotherms revealed that a 5 μm thick coating composed of hexane-derived pp-C:H NPs prepared at 100 W exhibits a specific surface area of 21.8 ± 2.5 m^2^ g^–1^, which is nearly double that of an equally thick coating prepared at 30 W (13.9 ± 1.8 m^2^ g^–1^; see the Supporting Information for measurement detail). In contrast, the interparticle void volume showed no statistically significant difference between the samples, reaching values of 0.012 ± 0.002 cm^3^ g^–1^ and 0.011 ± 0.001 cm^3^ g^–1^, respectively. This indicates that the smaller voids formed among the more numerous, smaller 100 W NPs collectively result in a total volume comparable to that of the larger voids between the fewer, larger 30 W NPs.

Figure 2c provides the mean NP sizes obtained from the statistical analysis of their size distributions. In accordance with previous research,^49^ a higher power produces smaller pp-NPs regardless of the monomer used because stronger molecular fragmentation in the plasma creates more nucleation centers (radicals and, to a lesser extent, ions), leading to a greater number of smaller pp-NPs. At a lower power, a smaller number of larger pp-NPs are formed.

The difference in the pp-NP size is also dependent on the type of monomer: the effect is particularly pronounced for the aliphatic monomers and less prominent for benzene, with pp-hexane NPs exhibiting a manifold disparity between the 30 and 100 W samples, whereas this difference for pp-benzene is only 2-fold. Simplified mechanistic considerations similar to those proposed earlier^35^ reveal that hexane has the highest sum of bond enthalpies (78 eV/molecule, Figure 1), which is close to the 87 eV mean energy supplied per monomer molecule at 30 W. Hence, of all the monomers polymerized at 30 W, hexane is closest to the power-deficient regime, in which larger molecular fragments at lower concentrations can be available for accretion of pp-NPs. At 100 W, the mean energy of 290 eV/molecule by far exceeds the sum of the monomer’s bond enthalpies, shifting the plasma polymerization toward the conditions of Yasuda’s “atomic polymerization”,^50^ at which monomer molecules are so heavily fragmented that high concentrations of very small fragments or even atoms are created in the gas phase to serve as nucleation centers and building blocks for the polymerization. As a result, an almost order-of-magnitude difference in the pp-NP size can be achieved when increasing the power from 30 to 100 W for hexane.

Other monomers also followed this trend. Cyclohexane has a lower sum of bond enthalpies (73 eV/molecule) compared to hexane, and benzene has the lowest value (56 eV/molecule), which puts benzene further into the monomer-deficient regime, even for 30 W. As a result, the difference in the pp-benzene NP size is not as large as that for aliphatic monomers. Nevertheless, there is still a 2-fold difference between 30 and 100 W, leaving some room to tune the porosity of the pp-NP deposits, even in the benzene case. Thus, a careful choice of the monomer and plasma parameters allows for fine-tuning of the pp-NP size distribution, which can be useful in applications where tunable porosity in the tens to hundreds nanometer range is of primary concern.

The main mediators of plasma polymerization, free radicals, are quenched by recombination processes, although a significant fraction remains unquenched and is trapped within the plasma polymer matrix. This is evident from ESR spectroscopy that shows the presence of spin signals in all pp-NPs (Figure 3). For all monomers, the spin concentration decays over time, indicating postdeposition quenching of radicals by oxygen in air. Notably, if the three monomers are compared together, there is a clear correlation between the monomer structure and the concentration of free radicals. Aliphatic pp-hexane NPs contain the smallest number of free radicals, while aromatic pp-benzene NPs contain the highest concentration of free radicals.

**Figure 3:**
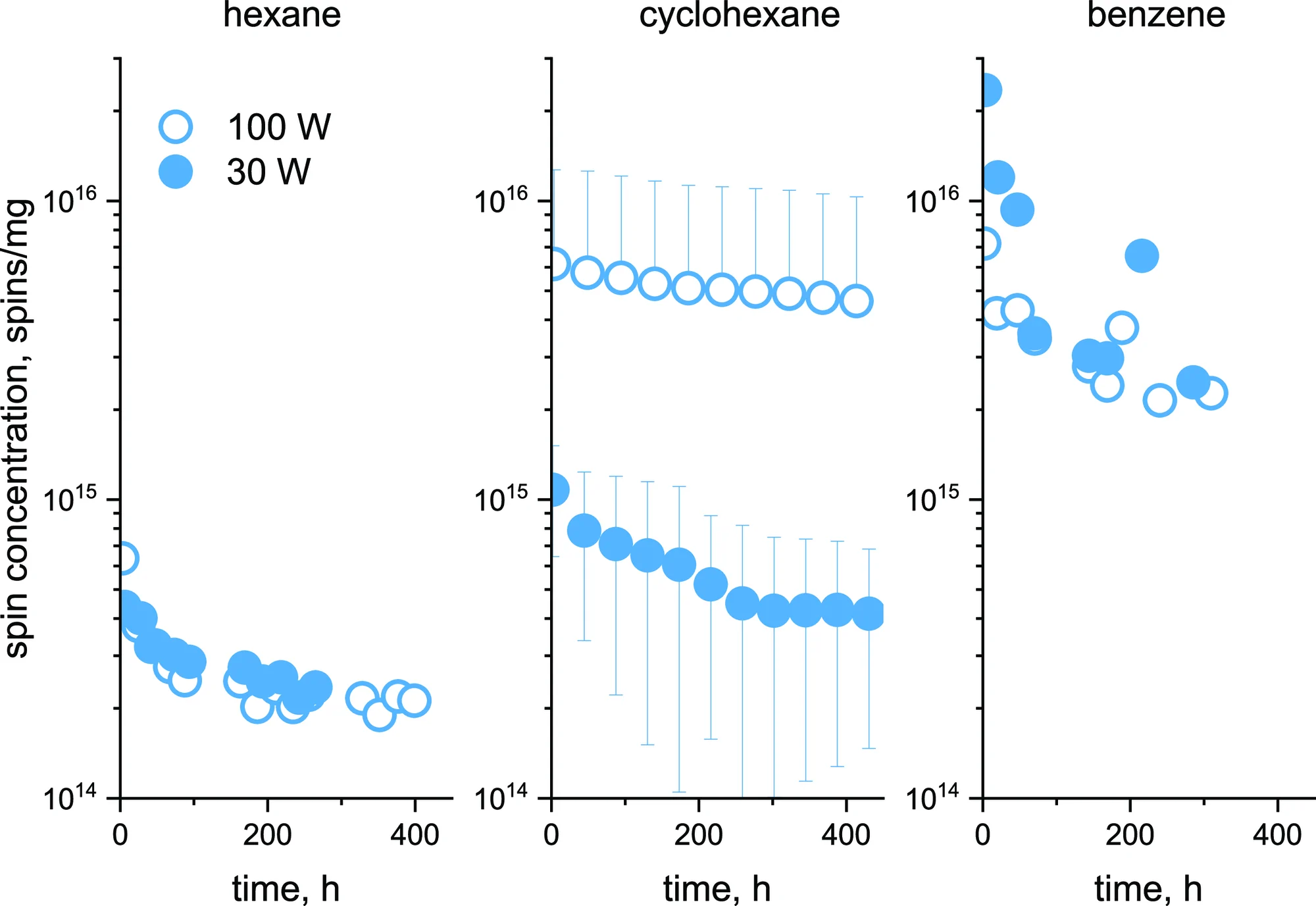
Decay of spin concentration in pp-NPs prepared from different monomers at 30 and 100 W and stored in air. Time zero denotes the first acquired data point, obtained 1 h after sample preparation.

In hydrocarbon plasmas, radicals form due to electron-impact-driven hydrogen detachment, opening of unsaturated bonds, and cleavage of C–C bonds.^51^ Hydrogen detachment is followed by the recombination of atomic hydrogen, forming H_2_, which escapes the discharge zone without participating in the polymerization processes. Therefore, this radical-quenching channel reduces the number of trapped radicals and is most efficient for linear aliphatic molecules with many hydrogen atoms, such as hexane.^51^ For benzene, the number of hydrogen atoms per molecule is lower and hydrogen detachment is less prominent. Furthermore, ring opening creates biradicals, thereby increasing the overall radical concentration.^52^ Therefore, the number of unquenched radicals in pp-benzene NPs is also higher.

Regarding power dependence, no significant difference is found between the 30 and 100 W samples of pp-NPs. NPs of pp-cyclohexane may show some indications of a higher radical concentration in the 100 W sample, but the results are strongly influenced by statistical error arising from large uncertainties in determining the sample mass and the spin concentration.^53^ The relatively low deposition rates of pp-NPs, and consequently the small sample masses, leave the power dependence as an open question that requires further investigation. Overall, the ability to tune the radical concentration in pp-NPs can be beneficial for biomedical applications that consider linker-free radical-driven biomolecule binding.

The loss of hydrogen due to hydrocarbon bond cleavage in the monomer molecules is further evidenced by ERDA performed on the pp-NPs. For all monomers, plasma polymerization reduces the hydrogen content of pp-NPs (Figure 4 and Table 1).

**Figure 4:**
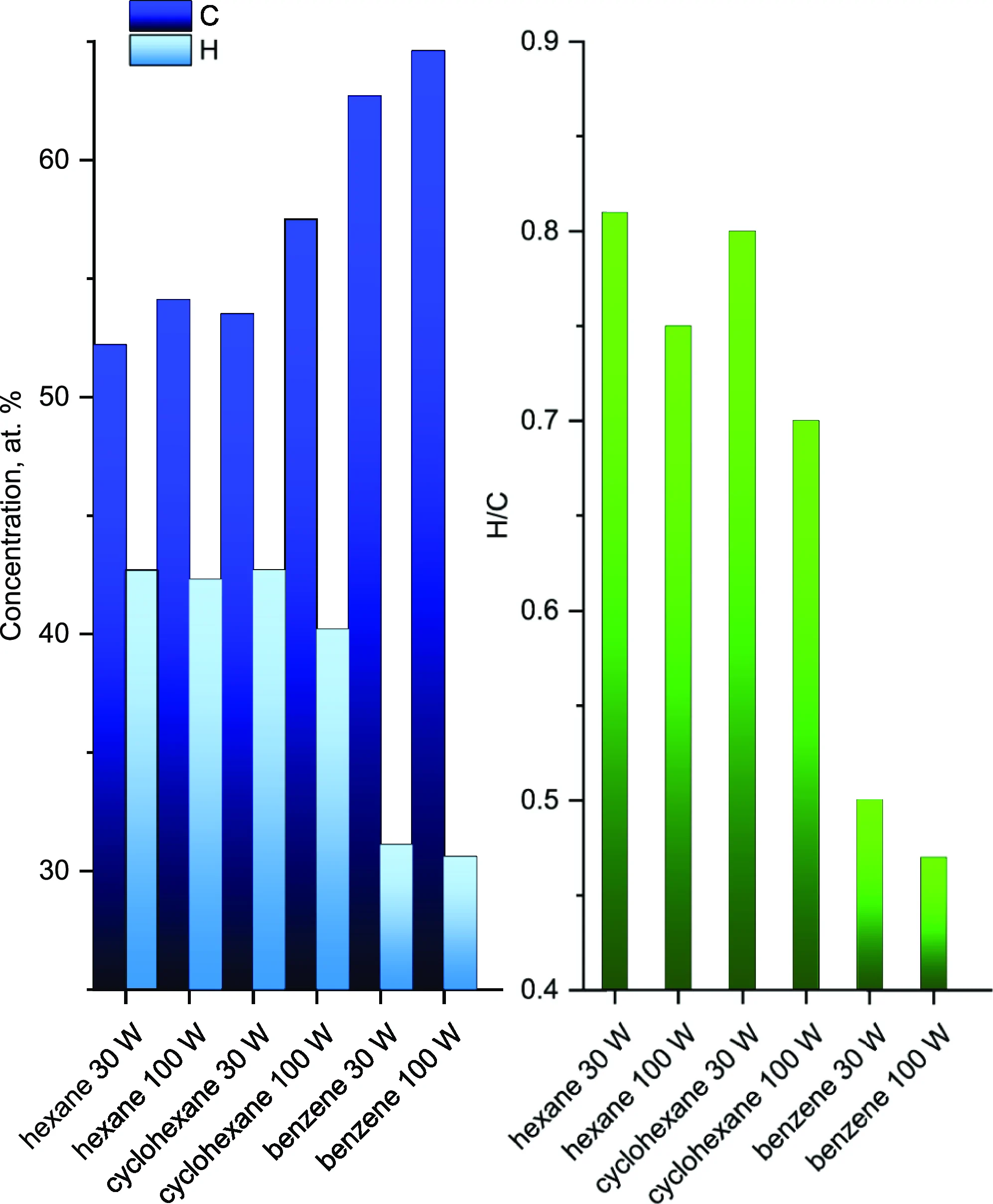
Carbon and hydrogen contents of pp-NPs prepared from different monomers at 30 and 100 W, as determined by ToF-ERDA analysis.

**Table 1:** Total Elemental Content of pp-NPs as Determined by ToF-ERDA Analysis, along with the Corresponding Values Calculated for the Monomers

	elemental concentration, at. %					
sample	C	H	O	N	H/C	Hplasmapolymer/CHmonomer/C
hexane 30 W	52	43	4	1	0.83	0.36
hexane 100 W	54	42	4	0	0.78	0.33
*monomer hexane*	*30*	*70*	-	-	*2.33*	-
cyclohexane 30 W	53	43	3	1	0.81	0.41
cyclohexane 100 W	58	40	2	0	0.69	0.35
*monomer cyclohexane*	*33*	*67*	-	-	*2.00*	-
benzene 30 W	63	31	5	1	0.49	0.49
benzene 100 W	65	30	5	0	0.46	0.46
*monomer benzene*	*50*	*50*	-	-	*1.00*	-

Since all experiments are performed in the monomer-deficient regime, the difference in hydrogen retention between 30 and 100 W is not drastic. Nevertheless, a general trend emerges: higher power is more destructive, resulting in lower H/C ratios across all monomers. To further examine the effect of discharge power, additional experiments were performed for hexane-derived pp-C:H NPs at intermediate powers (45 and 60 W), and the results are presented as Figure S8 in the Supporting Information. A clear monotonic trend is observed: increasing power leads to a pronounced decrease in the NP size, accompanied by a gradual reduction in the H/C ratio.

In the hexane–cyclohexane–benzene sequence, hexane undergoes the greatest hydrogen loss, as its H/C ratio decreases 3-fold compared to that of the monomer. Cyclic monomers, especially aromatic benzene, experience a less dramatic hydrogen loss; the H/C ratio of the latter shows only a 2-fold decrease. However, in terms of absolute atomic concentrations, the aliphatic monomers provide the highest hydrogen retention, approaching an equiatomic H/C ratio. This can be particularly important for the application of pp-NPs as nanofuels for laser-driven proton-boron fusion, where high hydrogen retention is crucial.^30,31,54^

To verify how the plasma polymerization pathways manifest in the chemical structure of the pp-NPs, we analyzed their FTIR spectra (Figure 5 and Table 2). For reference, oligoethylene and oligostyrene films were prepared by vapor-phase deposition, as described in our previous works.^9,55,56^ The reference FTIR spectra demonstrate well-resolved stretching and deformation bands of aliphatic CH_2_ groups at 2918, 2850, 1462, and 1373 cm^–1^. In the case of oligostyrenes, these are also complemented by stretching and deformation modes of aromatic CH groups at 3081, 3059, and 3026 cm^–1^, monosubstituted benzene ring fingers in the range of 1946–1728 cm^–1^, and stretching modes of aromatic carbon–carbon bonds (ring modes) at 1600, 1495, and 1452 cm^–1^. Regardless of the monomer used, the FTIR spectra of ppNPs are less resolved, reflecting the diversification of chemical motifs in plasma polymers.^6,13,57^ Nevertheless, several trends can be identified. Stretching and deformation bands of the aliphatic CH_2_ groups remain present but become enriched with contributions from CH and CH_3_ groups, which justifies the CH_*x*_ label in the spectra. Furthermore, new bands appear in pp-NPs that are associated with radical quenching via recombination, leading to the formation of unsaturated carbon bonds (C=C at 1655–1600 cm^–1^ and C≡C at 2240–2180 cm^–1^), or via postdeposition reactions with atmospheric oxygen-containing molecules, resulting in the formation of hydroxyl groups centered at 3400 cm^–1^ and carbonyl-based moieties in the range of 1720–1700 cm^–1^. For benzene-derived pp-NPs, the aromatic signatures become substantially attenuated with respect to the aliphatic vibrations, evidencing the partial destruction of benzene rings.

**Figure 5:**
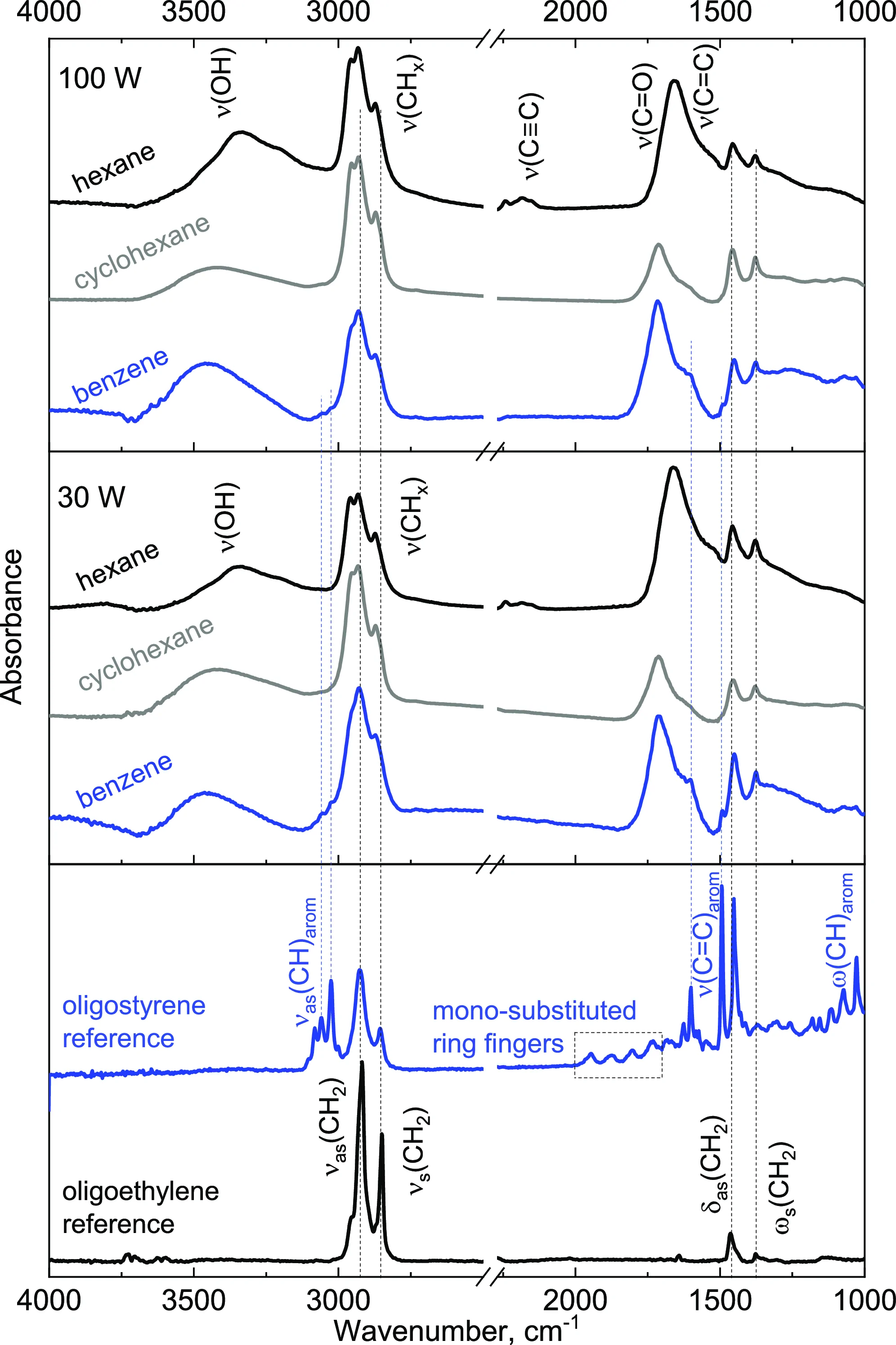
FTIR spectra of pp-NPs prepared from different monomers at 30 and 100 W, shown together with reference spectra of uncross-linked oligoethylenes and oligostyrenes.

**Table 2:** Assignment of FTIR Bands for pp-NPs Deposited from Different Monomers, Together with Reference Spectra of Uncross-Linked Oligoethylenes and Oligostyrenes^58,59^

wavenumber, cm^–1^	vibration
3400	ν (OH), pp-NPs
3081, 3059, 3026	ν (CH)_aromatic_, oligostyrenes
2918, 2850	ν_as_ (CH_2_), ν_s_ (CH_2_), oligostyrenes, oligoethylenes
2240, 2180	ν (C≡C), pp-NPs
1946, 1876, 1801, 1728	benzene ring fingers, summation bands, oligostyrenes
1720–1700	ν (C=O), pp-NPs
1655–1600	ν (C=C), pp-NPs
1600, 1495, 1452	ν (C=C)_aromatic_, ring modes, oligostyrenes
1462, 1373	δ_as_ (CH_2_), ω_s_ (CH_2_), oligoethylenes
1028	ω (C–H)_aromatic_, oligostyrenes

Regarding power dependence, the spectra of hexane- and cyclohexane-derived ppNPs at 30 and 100 W are very similar, indicating comparable chemical composition. The slightly stronger dehydrogenation at 100 W, as detected by ToF-ERDA, cannot be reliably distinguished in the FTIR spectra. For benzene-derived pp-NPs, the aromatic signals at 3081–3026 cm^–1^ and 1655–1600 cm^–1^ are stronger for 30 W as compared to 100 W, indicating better retention of benzene rings at lower power. This finding is also consistent with complementary XPS measurements (Figure S9 of the Supporting Information), which show a stronger shakeup satellite (a marker of the π–π* excitation in aromatic systems) in the C 1s spectrum of the 30 W benzene sample.

Structural peculiarities of the pp-NPs were studied by VEPALS, which is uniquely sensitive to angstrom-scale free volumes in polymers. A complete set of the five lifetime components (τ_1_–τ_5_) with the corresponding intensities (*I*_1_–*I*_5_) obtained from the VEPALS spectra is given in Figures S5–S7 of the Supporting Information for pp-hexane, pp-cyclohexane, and pp-benzene NPs; here, we focus only on the (τ_3_, *I*_3_) component. The τ_3_ is found at about 2 ns, a time scale typically attributed to free volumes in polymers.^60,61^ For comparison, VEPALS data were acquired for a reference thin film of oligoethylenes, which are mixtures of uncross-linked (CH_2_)_*x*_ oligomers characterized by a mean molar mass of *M*_n_ = 1.4 × 10^3^ g/mol and dispersity of *D*_M_ = 1.10.^55,56^ The τ_3_ component shows constant values in the kinetic energy range *E* = 2–5 keV for oligoethylenes (Figure 6). At lower and higher energies, the results are affected by surface roughness and by the fraction of positrons reaching the substrate. Therefore, the kinetic energy range of 2–5 keV was selected for the following analysis of the VEPALS results.

**Figure 6:**
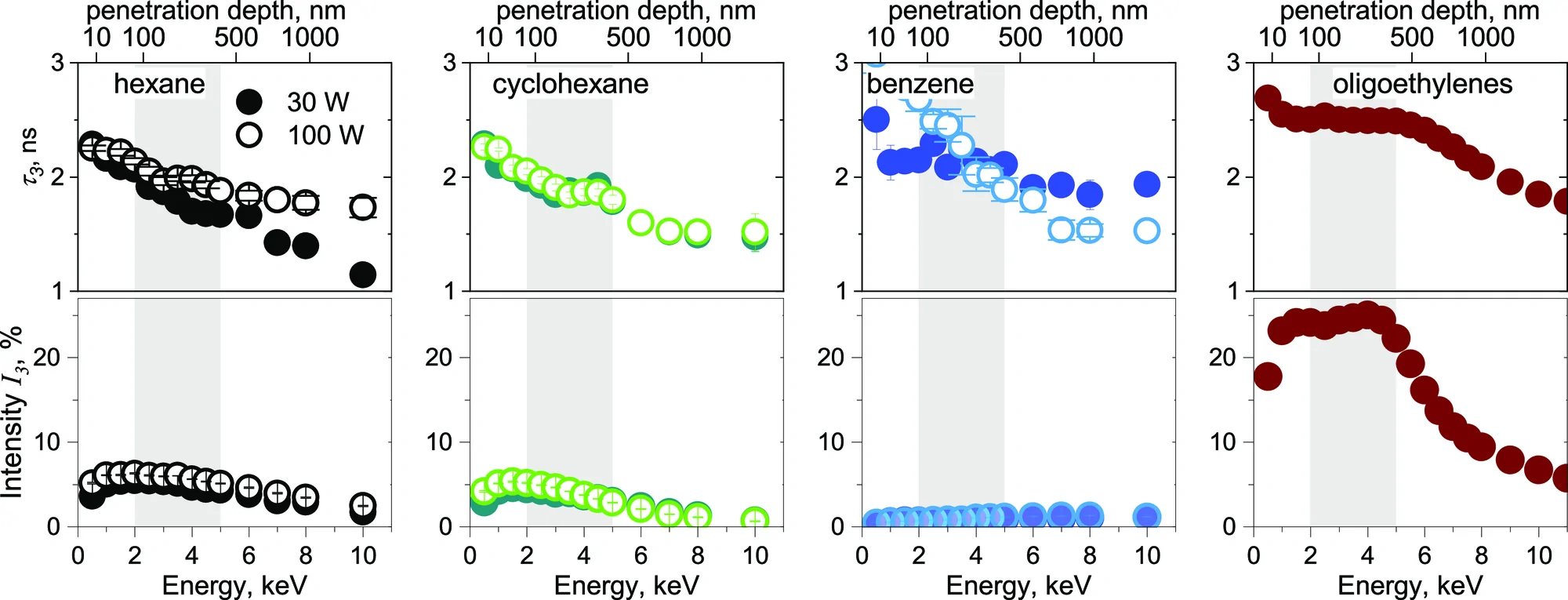
Characteristic VEPALS lifetime τ_3_ and its relative intensity *I*_3_ arising from free volumes within pp-NPs prepared from different monomers and in a (CH_2_)_100_ oligoethylene film prepared by vacuum thermal evaporation, plotted as a function of the incident positron energy. Solid and open symbols correspond to samples prepared at 30 and 100 W, respectively. The mean positron penetration depth is shown on the upper *x*-axis. Shaded areas indicate the region of the deposits least influenced by surface roughness or the substrate.

Even within this range, τ_3_ and *I*_3_ show deviations from constant values, which we attribute to substrate-associated artifacts manifested in highly porous material. Nevertheless, it can be observed that the overall positions of the τ_3_ = *f*(*E*) curves shift to larger values in the sequence hexane–cyclohexane–benzene, while the intensity *I*_3_ decreases. This trend indicates that the mean size of free volumes increases, whereas their concentration decreases along this sequence. For uncross-linked oligoethylenes, the τ_3_ component, and especially its intensity *I*_3_, notably exceeds those of the pp-NPs, confirming a strongly cross-linked nature of the plasma polymer matrices.

Using the extended Tao-Eldrup model,^62–64^ we calculated the mean diameters of free volumes *d*_3_ in the energy range *E* = 2–5 keV and compared these values for different monomers and powers (Figure 7a). For pp-hexane and pp-cyclohexane NPs, the resulting *d*_3_ values remain at approximately 0.55 nm, which is smaller than those observed for pp-benzene NPs. In the latter case, not all benzene rings are destroyed in the plasma, and their incorporation into the plasma polymer matrix induces steric effects that preserve larger unoccupied spaces around the aromatic moieties. Nevertheless, the concentration of free volumes (*I*_3_) is lowest for pp-benzene NPs, indicating tighter molecular packing in the nonaromatic regions of the matrix. A schematic illustration emphasizing this contrast is listed in Figure 7a. For all pp-NPs, both the size and concentration of free volumes are lower than those in uncross-linked oligoethylenes (*d*_3_ = 0.67 nm), pointing to a high cross-link density in the plasma polymers.

**Figure 7:**
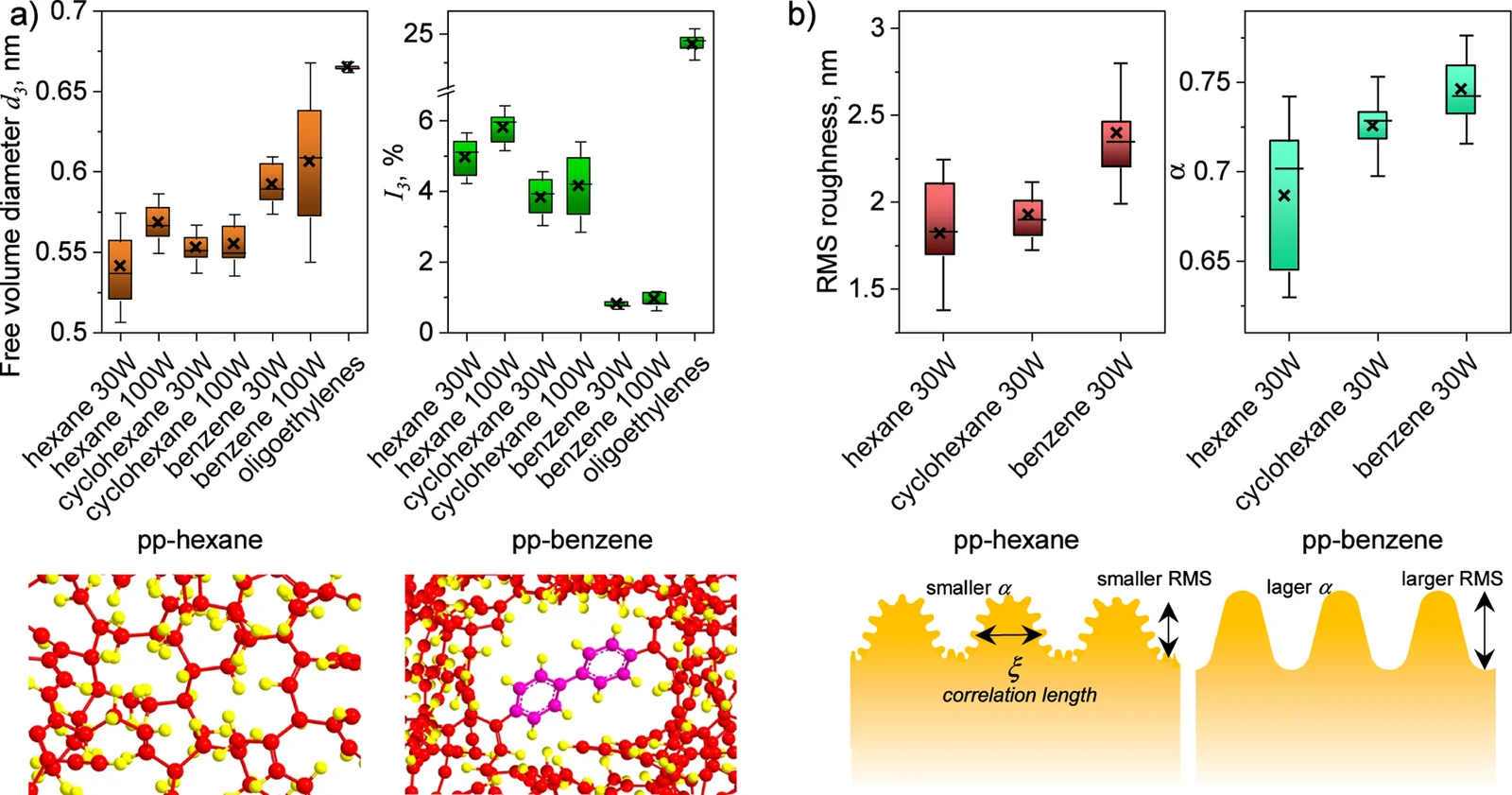
(a) Mean free-volume diameters extracted from τ_3_ and the corresponding mean intensities *I*_3_ obtained from VEPALS measurements in the range *E* = 2–5 keV for ppC:H NPs prepared from different monomers at different powers, and for a (CH_2_)_100_ oligoethylene film prepared by vacuum thermal evaporation (at the 0.05 significance level, population means are significantly different (ANOVA)); (b) mean RMS roughness and roughness exponent α of the surfaces of pp-NPs prepared at 30 W (error bars represent standard deviations; at the 0.05 significance level, population means for pp-benzene NPs are significantly different from those for pp-hexane and pp-cyclohexane NPs, ANOVA).

It is also worth noting that pp-NPs prepared at higher power exhibit larger *d*_3_ and *I*_3_ values, indicating an increase in free-volume size and concentration. This observation suggests a lower effective cross-link density at 100 W, which may appear counterintuitive, as thin films of plasma polymers typically become more cross-linked with increasing power. We propose that this opposite behavior in pp-NPs arises from the fundamentally different charging conditions experienced by NPs compared with supported thin films grown under floating-potential conditions.

Specifically, at 100 W, the discharge generates a higher concentration of pp-NPs, possibly leading to a regime of high dust density where the mean interparticle distance becomes sufficiently small for collective effects to dominate. Under such conditions, the system may approach the “electron-depleted” regime described by the Havnes effect, in which individual particle sheaths overlap, and a collective cloud potential is formed.^15,18^ In this regime, the net charge per NP is significantly reduced compared to isolated NPs, resulting in weaker ion bombardment. Experimental evidence for such behavior has been reported in dusty plasma systems generated in argon–acetylene discharges,^18^ where plasma diagnostics based on dust density waves, extinction measurements, and Mie polarimetry demonstrate strong electron depletion (Havnes parameter *P* ≫ 1) and consequently very low NP charges despite high NP densities. In these conditions, the NP charge is governed primarily by electron depletion rather than by local plasma parameters and can be reduced by more than an order of magnitude compared to isolated particles.

By analogy, such reduced charging in a dense pp-NP cloud would imply less energetic ion bombardment of the growing NPs, leading to reduced bond scission and network rearrangement during growth and therefore to a lower cross-link density, consistent with the increased free-volume size and concentration observed by VEPALS at 100 W.

It should be emphasized, however, that while this interpretation is supported by experimental observations in analogous dusty plasma systems, direct plasma diagnostics (e.g., NP charge, electron density, or Havnes parameter) have not yet been performed under the specific conditions of the present study. Therefore, the proposed mechanism should be regarded as a plausible hypothesis that requires further experimental validation rather than as a definitive explanation.

To explore the surface topology of the pp-NPs, AFM imaging with a supersharp probe was employed. The pp-NPs prepared at 30 W were sufficiently large to allow scanning of the upper hemispheres of their surfaces over an area of 200 × 200 nm, enabling quantitative analysis in terms of the RMS roughness and height–height correlation functions. A series of individual 30 W pp-hexane, pp-cyclohexane, and pp-benzene NPs were scanned in this manner (Figures 2a and S1 of Supporting Information). The RMS roughness values were used directly as obtained, while the height–height correlation functions were plotted in log–log coordinates, and the roughness exponent α was derived from the slope of their linear regions, as shown in Figure S2 of the Supporting Information. The RMS roughness and α values were averaged using the ANOVA protocol to compare their means (Figure 7b).

It is interesting to note that lower RMS roughness values obtained for pp-hexane NPs correlate with lower α values, whereas higher RMS roughness values observed for pp-benzene NPs correlate with higher α values. The data for pp-cyclohexane NPs lie between those of the other two monomers. The RMS roughness provides a global measure of surface profile fluctuations beyond the lateral correlation length, while the correlation length defines the characteristic lateral size of surface protrusions and depressions. Thus, the roughness exponent α describes the short-range roughness of the surface at length scales below the correlation length, with larger α values indicating smoother local surface profiles, whereas smaller α values correspond to more jagged local surface profiles.^65,66^ Accordingly, the local landscape of the surface features of pp-hexane NPs is more jagged than that of pp-benzene NPs. A schematic illustration emphasizing this difference is provided in Figure 7b.

Further insight into cross-link density in pp-NPs can be obtained by performing statistical reconstruction of plasma-polymer network structure using the framework of the TBP.^41–45^ By definition, a cross-link represents a junction point that supports at least three infinite network pathways, while an EANC is defined statistically as a polymer segment linking two cross-links. In this study, the BUs were constructed from carbon and hydrogen atoms and, for benzene, intact benzene rings, reflecting experimental evidence that aromatic motifs partially survive plasma polymerization. Connectivity among BUs was represented through a restricted set of OLs corresponding to single, double, and triple carbon-to-carbon bonds, as well as C–H bonds, leading to a finite set of 19 distinct BUs (Figure 8). The relative molar concentrations of BUs were encoded in a generating function and served as the statistical input to TBP (see Supporting Information for details).

**Figure 8:**
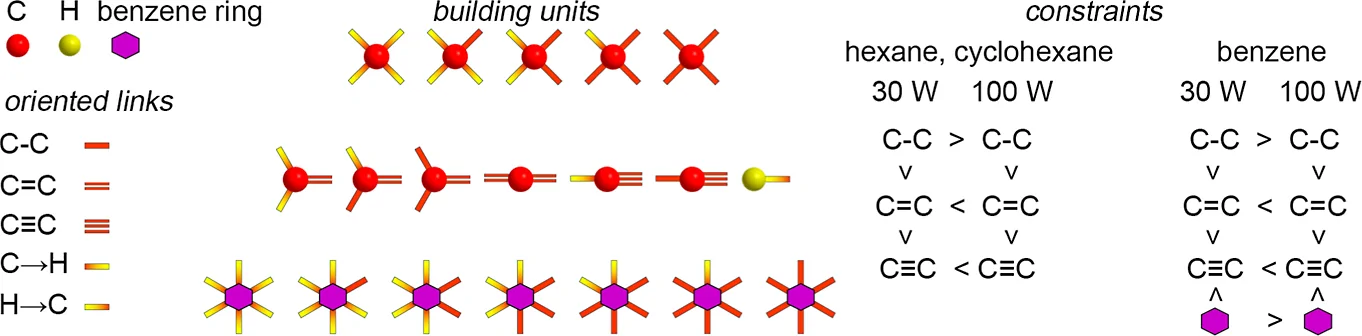
BUs, OLs, and empirical constraints imposed on the ratio between carbon–carbon bond types used to describe connectivity in the statistical reconstruction of the pp-NP network structure.

The experimentally determined elemental H/C ratios (Table 1) for hexane-, cyclohexane-, and benzene-derived pp-NPs prepared at 30 and 100 W were used to specify the relative molar concentrations of all resulting BUs. In addition, qualitative spectroscopic signatures of selected functional motifs were used to apply further constraints (Figure 8). For example, the concentration of single C–C bonds was assumed to be substantially higher than that of C=C bonds, whereas the concentration of C=C bonds was assumed to be higher than that of C≡C bonds. This assumption is supported by generalized evidence from hydrogenated amorphous carbon, which represents the closest analogue to hydrocarbon plasma polymers.^67^

In the case of pp-benzene, only a minor fraction of benzene rings survived in plasma polymerization; therefore, their concentration in the resulting plasma polymers was assumed to be significantly lower than that of single C–C bonds (see FTIR spectra in Figure 5). Nevertheless, benzene rings were assumed to remain more abundant than C≡C bonds and to be present at levels comparable to C=C bonds.

Regarding the dependence on discharge power, constraints were imposed such that unsaturated carbon bonds become slightly more abundant at higher power. This follows from FTIR spectra, which show a small increase in C≡C bonds for hexane and do not provide a reliable trend for C=C bonds. Benzene rings exhibit the opposite trend, as their concentration decreases with increasing power according to both FTIR and XPS analyses. All of these constraints were incorporated into the model.

Furthermore, the analysis is carried out within the sol + gel scenario, in which pp-NPs contain both a cross-linked gel (infinite network) and a sol fraction. This scenario appears realistic, as plasma polymerization may result in incomplete gelation, leaving a fraction of low-molar-mass components embedded within the plasma polymer structure.^8,9,68–71^ Nevertheless, as shown in the Supporting Information, both limiting descriptions (sol + gel and gel only) produce identical trends across the investigated systems, differing only in the absolute magnitude of the elastic network density. For this reason, and because the sol + gel description provides the most direct link to the experimentally accessible composition, the sol + gel scenario is adopted throughout the discussion.

Table 3 shows an illustrative TBP-based reconstruction of the plasma polymer network, using a representative set of assumed C–C, C=C, and C≡C bond fractions and benzene-ring content that satisfy the imposed constraints. This example is nonunique and is intended to demonstrate the response of the TBP framework to plausible input parameters rather than to provide a definitive structural description.

**Table 3:** Example Calculations of the Concentration of EANC in pp-NPs Prepared from Different Monomers at 30 and 100 W, as Determined by Statistical Reconstruction within the TBP Framework

	pp-hexane		pp-cyclohexane		pp-benzene	
	30 W	100 W	30 W	100 W	30 W	100 W
Percentage of C–C Bond Types in pp-NPs (Hypothesized)						
C–C	80	70	80	70	92	88
C=C	15	20	15	20	6	9
C≡C	5	10	5	10	2	3
Ratio of Intact Benzene Rings in pp-NPs Relative to Benzene Monomer (Hypothesized), *r*_benz_						
	-	-	-	-	0.05	0.04
Number of EANC per C Atom, *n*_e_						
gel + sol	0.728	0.465	0.745	0.531	1.326	1.236

The magnitude of the EANC concentration reflects the distance from the gel point. An upper bound on the network density can be estimated from the maximal number of elastically active chains that can emanate from each structural unit. If each carbon atom contributes up to four chain segments, and each benzene ring contributes up to six chain segments shared among six carbons, the maximal number of EANCs per carbon atom (including carbons in benzene rings) simplifies to

$$n_{e}=2-\frac{3}{2}r_{benz}$$

Here, *r*_benz_ quantifies the fraction of benzene rings that remain intact after plasma polymerization. For pp-hexane and pp-cyclohexane, the upper bound is *n*_e_ = 2, while for pp-benzene, 30 W and 100 W, it equals 1.93 and 1.94, respectively. The deeper the system lies in the gel region, the closer *n*_e_ is to the upper bound limit.

Within the assumptions, the results in Table 3 demonstrate that *n*_e_ increases in the sequence hexane–cyclohexane–benzene, with only minor differences between the aliphatic monomers and a substantially larger increase for benzene. Given that, for pp-benzene, *n*_e_ is closer to the upper bound limit than for the aliphatic plasma polymers, pp-benzene is positioned furthest beyond the gel point, exhibiting the lowest sol content and the highest cross-link density. This behavior follows the VEPALS-measured decrease in free-volume concentration across the same sequence. Considering that C–H bonds terminate chain propagation, the intrinsically lower hydrogen content in benzene than in the aliphatic monomers increases the probability of carbon–carbon coupling, branching, and cross-linking in the nonaromatic part of the matrix. However, a fraction of benzene rings survives plasma polymerization and becomes incorporated into the network; their large, planar geometry restricts local packing and generates larger intrinsic voids in their vicinity. This interpretation is also consistent with the larger free volumes observed in pp-benzene NPs and the smaller free volumes in pp-hexane and pp-cyclohexane NPs. However, these TBP trends depend on hypothesized bond-type distributions that are not independently quantified and therefore should not be interpreted as direct experimental validation.

A decrease in *n*_e_ at higher discharge power is consistently obtained when a higher fraction of unsaturated bonds is assumed. This provides a mechanistic scenario consistent with spectroscopic indications of increased unsaturation but remains conditional on the selected parameter space. Since the *n*_e_ values strongly depend on the unknown C–C/C=C/C≡C ratios, the TBP model identifies ranges of physically plausible inputs that reproduce the experimentally observed trends rather than establish a unique structural solution. More details of the *n*_e_ behavior for pp-hexane, pp-cyclohexane, and pp-benzene are provided in the Supporting Information.

To perform a more general analysis with fewer assumptions, the number of parameters was reduced by neglecting contributions from C≡C bonds, and networks were reconstructed for arbitrary combinations of H/C, C=C/C–C, and *r*_benz_. Figure 9 shows 3D contour plots of *n*_e_ ranging from weakly cross-linked (*n*_e_ = 0.4) to highly cross-linked (*n*_e_ = 1.6) matrices. All relevant parameters are treated as variables, so the plot is not tied to any particular system but instead represents general trends in hydrocarbon plasma polymer matrices.

**Figure 9:**
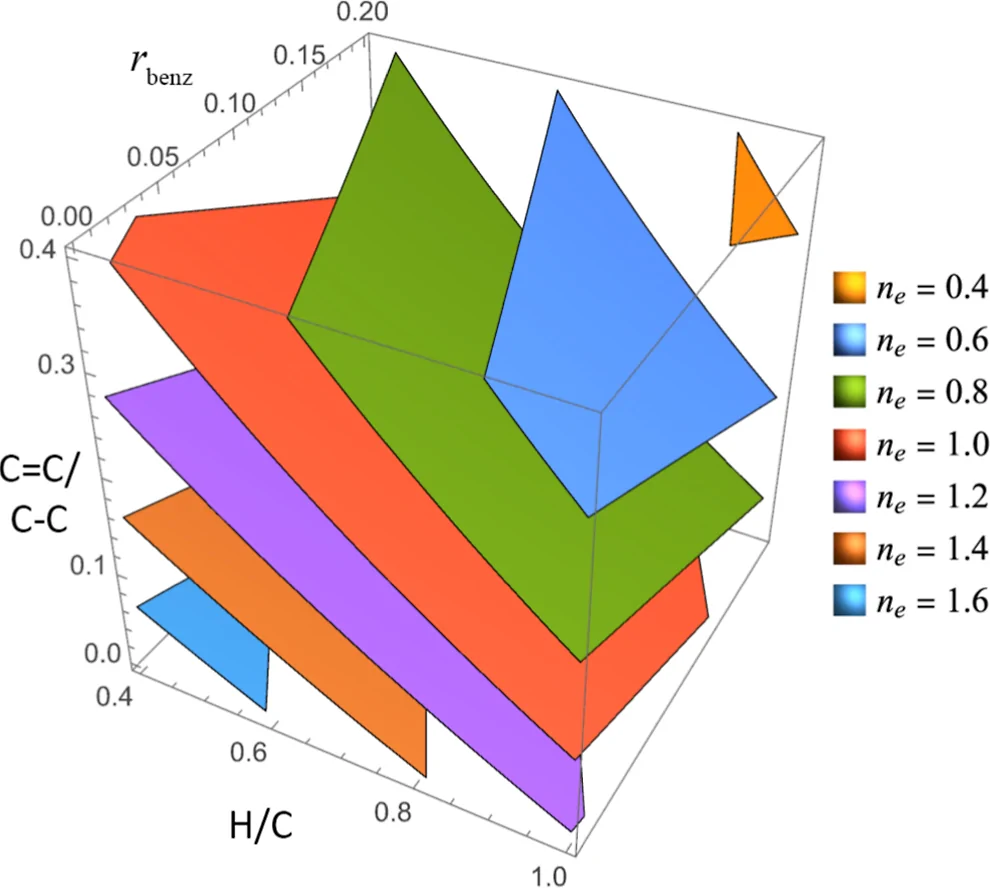
Contour 3D plots of the number of EANC per carbon atom in plasma polymer matrices as a function of the H/C ratio, the C=C/C–C ratio, and the benzene-ring retention *r*_benz_ (C≡C bonds neglected).

Dehydrogenation (lower H/C) increases the functionality of network nodes, thereby promoting higher network density. Conversely, an increase in unsaturated bonds (higher C=C/C–C) reduces node functionality, increases extinction probabilities, and thus decreases the network density. These two effects therefore counteract each other, and their interplay determines the resulting EANC concentration in plasma polymers. A stronger contribution from unsaturated bonds explains why the 100 W systems exhibit lower network densities than their 30 W counterparts and aligns with the experimentally observed higher free-volume counts and larger average free-volume diameters at 100 W. The incorporation of aromatic structures generally leads to a lower network density; however, this effect is less straightforward to interpret because *r*_benz_ is not independent of the H/C and C=C/C–C parameters, which are themselves linked to the fragmentation pathways of benzene rings.

Thus, the model may assist in predicting the resulting cross-link density in pp-NPs provided that independent quantification and tuning of the H/C, C=C/C–C, and *r*_benz_ ratios are possible. It should be explicitly stated that, while H/C ratios in plasma polymers can be reliably measured, quantitative information on the concentrations of C–C, C=C, and C≡C bonds, and aromatic structures is largely inaccessible with the current state of diagnostic techniques. This limitation represents the current bottleneck of the model for proper validation. Thus, the TBP approach cannot presently provide independent verification of cross-link density. It should instead be viewed as a predictive framework that rationalizes how different structural scenarios can give rise to the observed trends. In the future, quantitative tuning of these parameters via controlled experimental conditions, supported by model predictions, may enable ad hoc engineering of pp-NPs for advanced applications. For instance, the inner and outer nanostructural topologies of pp-NPs share key features with those of porous organic polymers (POPs).^72^ In particular, structural motifs in pp-benzene NPs show similarities to polymers of intrinsic porosity or porous aromatic frameworks, where microporosity arises from restricted conformational flexibility of aromatic BUs. In contrast, in pp-NPs, the structural design does not rely on specific multistep synthetic routes but instead emerges from a one-pot process driven by stochastic, nonequilibrium polyrecombination of monomer fragments.

Beyond these structural analogies, recent laser-driven proton–boron fusion experiments demonstrate the functional relevance of pp-NPs as nanostructured targets. Porous coatings of pp-hexanes NPs with sizes of 65 and 560 nm combined with boron-rich materials enable enhanced laser–matter interaction, leading to α-particle fluxes up to 5.6 × 10^8^ α sr^–1^ shot^–1^ and 5 × 10^7^ α sr^–1^ J^–1^, values competitive with the best reported results.^31^ These improvements are attributed to multiscale porosity, combining subnanometer free volumes with interparticle voids, which enhance laser absorption and energy conversion efficiency.

Given these shared characteristics with POPs and the demonstrated performance in laser-driven ion acceleration, pp-NPs may be promising for applications, such as selective gas adsorption and separation, environmental remediation, biomedical engineering, and energy storage and conversion.

## Conclusions

The present study demonstrates that the hierarchical structure of plasma polymer NPs is governed by a complex interplay between the monomer chemistry and plasma conditions. By comparing hydrocarbons with similar molar masses but different molecular structures (hexane, cyclohexane, and benzene), the roles of hydrogen content, bonding configuration, and fragmentation pathways in controlling radical retention, network formation, and nanoscale morphology are identified.

A central finding is that unquenched radical concentrations in the range of 10^14^–10^16^ spins mg^–1^ and free volumes of 0.5–0.6 nm emerge from the balance between radical generation, recombination, and steric constraints. Aromatic precursors promote higher radical concentrations and larger free-volume elements at lower concentrations, whereas aliphatic systems yield smaller yet more numerous free volumes, reflecting differences in hydrogen content and packing density.

Cross-link density is shown to be governed by competing mechanisms: dehydrogenation increases network connectivity, whereas unsaturated carbon bonds reduce the concentration of elastically active chains. This competition explains the observed decrease in effective cross-link density at higher discharge power (100 W), demonstrating that plasma energy input alone is not a sufficient predictor of the network structure.

Morphological analysis reveals scale-dependent surface characteristics, with aliphatic pp-NPs exhibiting lower global roughness but stronger local fluctuations (roughness exponent = 0.68), whereas aromatic pp-benzene NPs display higher global roughness and smoother local profiles (roughness exponent = 0.75). In addition, the NP size can be tuned from 65 to 560 nm, enabling control over interparticle voids in assembled coatings.

Overall, these findings establish a coherent framework linking plasma chemistry to structural organization across multiple length scales ranging from subnanometer free volumes to nanoscale morphology and mesoscopic porosity. This provides design guidelines for tailoring radical content, hydrogen retention, and multiscale porosity in plasma polymer NPs, supporting their further development for applications in drug delivery systems, energy-related technologies, and functional porous materials.

## Supplementary Material



## Notes

Details of AFM measurements; validation of ToF-ERDA; complementary hydrogen depth profiling by resonant NRA; N_2_ adsorption/desorption isotherm analysis; details of VEPALS measurements; discharge power dependence of the H/C ratio and NP size; XPS analysis; and details of statistical reconstruction of plasma polymer network structure (PDF)
