# Supplementary material for: Monomer Structure and Discharge Power Influence the Multiscale Architecture of Plasma-Polymerized Hydrocarbon Nanoparticles
Source: ACS Polym Au. 2026 Jun 16;6(5):1315–28. doi: 10.1021/acspolymersau.6c00072 (PMC13673887; doi:10.1021/acspolymersau.6c00072)
Supplement: Supplementary file 1 [file lg6c00072_si_001.pdf]

Supporting information

Monomer Structure and Discharge Power Influence Multiscale Architecture of Plasma-Polymerized Hydrocarbon Nanoparticles

Andrei Choukourov,[1*] Marco Tosca,[1,2] Ján Šomvársky,[1] Daniil Nikitin,[1] Mariia Protsak,[1] Iqra Wahid,[1] Jan Hanuš,[1] Veronika Červenková,[1] Ronaldo Katuta,[1] Krzysztof Tadyszak,[3] Jiří Pánek,[3] Eduardo Pitthan,[4] Dmitrii Moldarev,[4] Daniel Primetzhofer,[4] Jakub Čížek,[5] Eric Hirschmann,[6] Maciej Oskar Liedke,[6] Andreas Wagner,[6] Maik Butterling,[7] Hynek Biederman[1]

[1] Charles University, Faculty of Mathematics and Physics, Department of Macromolecular Physics, V Holešovičkách 2, 180 00 Prague, Czech Republic.

[2] ELI Beamlines Facility, the Extreme Light Infrastructure ERIC, Za Radnicí 835, 252 41 Dolni Brezany, Czech Republic.

[3] Institute of Macromolecular Chemistry CAS, Heyrovského nám. 2, 162 06 Prague, Czech Republic.

[4] Department of Physics and Astronomy, Ångström Laboratory, Uppsala University, Box 516, SE-751 20 Uppsala, Sweden.

[5] Charles University, Faculty of Mathematics and Physics, Department of Low Temperature Physics, V Holešovičkách 2, 180 00 Prague, Czech Republic.

[6] Institute of Radiation Physics, Helmholtz-Zentrum Dresden-Rossendorf (HZDR), Bautzner Landstr. 400, 01328 Dresden, Germany.

[7] Reactor Institute Delft, Department of Radiation Science and Technology, Faculty of Applied Sciences, Delft University of Technology, Mekelweg 15, NL-2629 JB Delft, The Netherlands

1. AFM

Super-sharp diamond-like carbon AFM tips with a guaranteed radius of 1 nm were used to scan sub-monolayers of pp-NPs prepared from hexane, cyclohexane, and benzene at a discharge power of 30 W (Figure S 1). Wide-area scans were first used to identify individual pp-NPs. High-resolution AFM images were then acquired by scanning the upper hemisphere of selected pp-NPs. The raw images were subsequently flattened by subtracting second-order polynomial backgrounds.

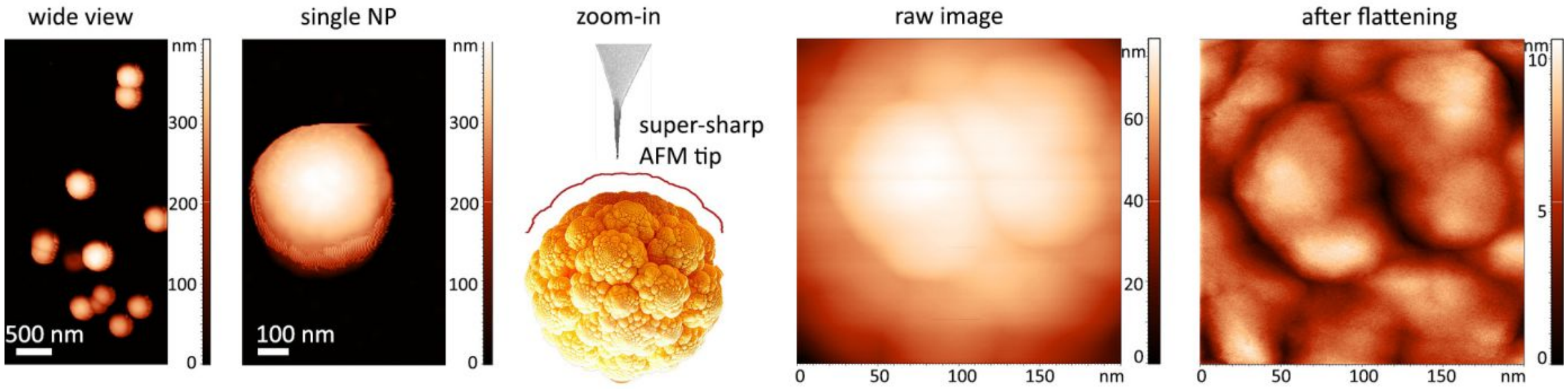

Figure S 1 Schematic of the AFM measurements performed with super-sharp tips on pp-NPs prepared from benzene at a discharge power of 30 W.

Height-height (HH) correlation functions were obtained from the flattened AFM images and plotted in log-log coordinates, as shown in Figure S 2. At lateral distances below the correlation length, the HH correlation functions follow a power law of the form $HH \sim r^{-2\alpha}$, where r is the lateral distance and α is the roughness exponent. The roughness exponent was therefore determined from the slope of the linear regions of the HH correlation functions. Fifteen pp-hexane, five pp-cyclohexane and fifteen pp-benzene NPs were analyzed to obtain the average values of the RMS roughness and the corresponding roughness exponent.

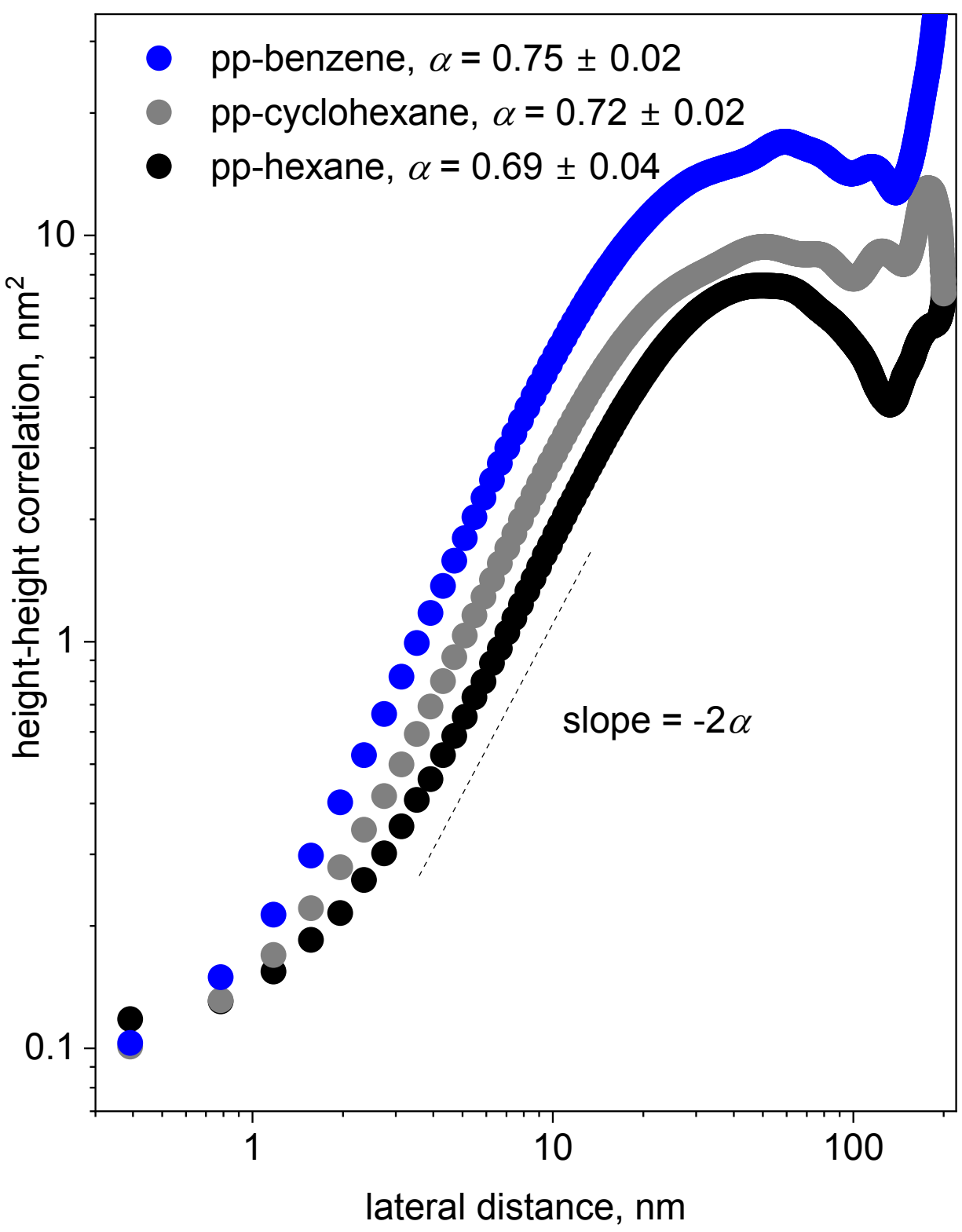

Figure S 2 Height-height correlation functions obtained from the 200×200 nm AFM surface scans of pp-NPs prepared from hexane, cyclohexane, and benzene at a discharge power of 30 W.

2. ToF-ERDA

In the ToF-ERDA measurements, exposure of the pp-NPs to $^{127}I^{8+}$, 36 MeV ion beam resulted in sample degradation accompanied by dehydrogenation, as evidenced by the evolution of the hydrogen-to-carbon ratio calculated for different beam exposure times (Figure S 3). To minimize measurement errors, depth profiles of the atomic concentrations were extracted from the first 50 seconds of acquisition for all samples.

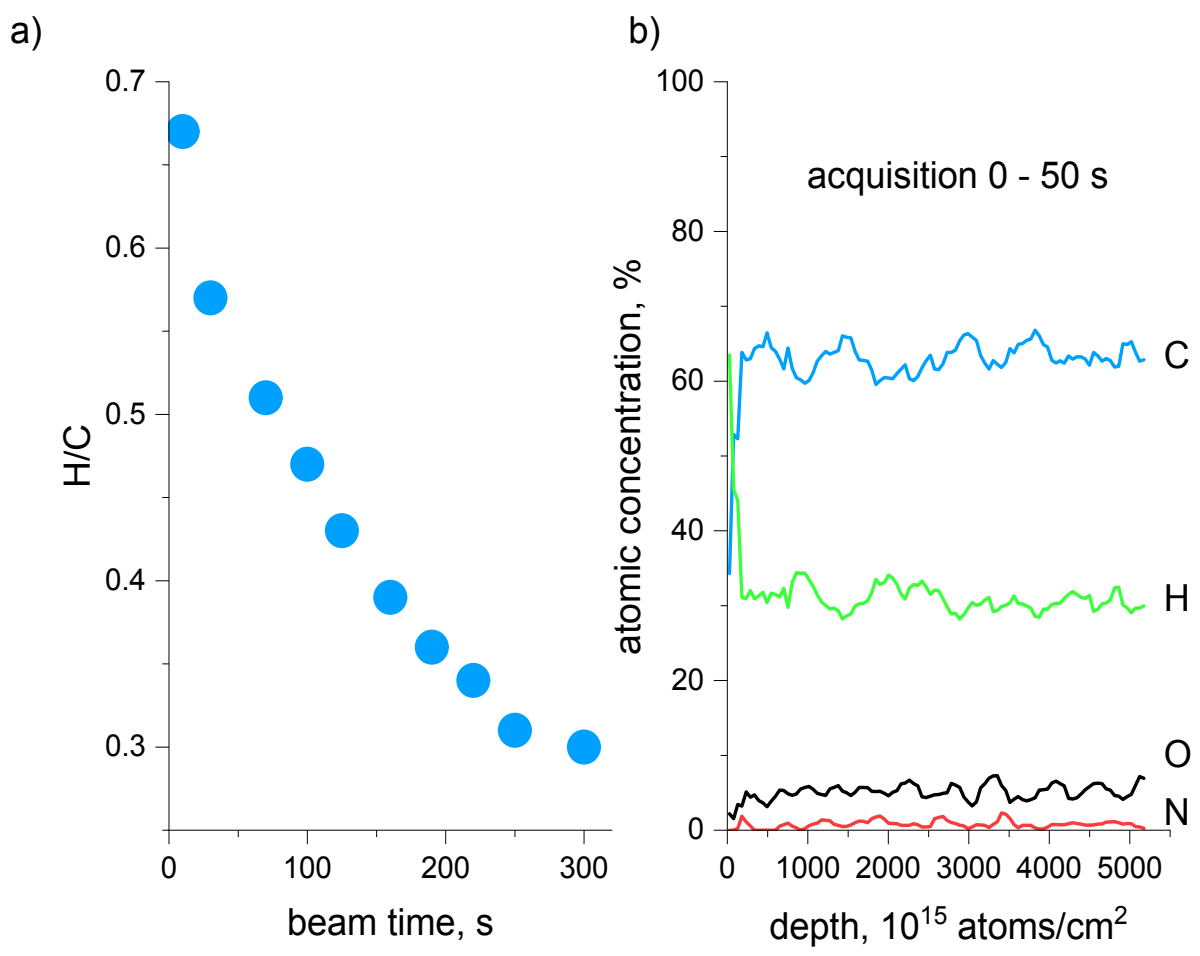

Figure S 3 Evaluation of the effect of the $^{127}I^{8+}$, 36 MeV ion beam on the acquired elemental content of pp-NPs prepared from benzene at a discharge power of 30 W: a) evolution of the hydrogen-to-carbon ratio as a function of beam exposure time; b) influence of acquisition time on the elemental depth profile.

For independent validation, hydrogen depth profiling using resonant Nuclear Reaction Analysis (NRA). This technique is based on the resonant nuclear reaction between hydrogen nuclei (^{1}H) in a sample and an incident ^{15}N ion beam tuned to a nuclear resonance energy of 6.385 MeV:

$$^{1}H + {}^{15}N \rightarrow {}^{12}C + \alpha + \gamma$$

The emitted γ-photons (4.43 MeV) were detected, and their intensity is directly proportional to the hydrogen concentration in a specific depth. By scanning the incident beam energy across the nuclear resonance, the depth profile of hydrogen in the sample can be determined. A representative sample (benzene-derived pp-C:H nanoparticles prepared at 30 W) was analyzed using this method (Figure S 4). The forward and backward scans reveal a slight hydrogen loss under ion irradiation, consistent with the behavior observed in ToF-ERDA measurements, although less pronounced. Importantly, NRA unambiguously confirms the presence of hydrogen, and the measured hydrogen concentration is in good agreement with the values obtained by ToF-ERDA.

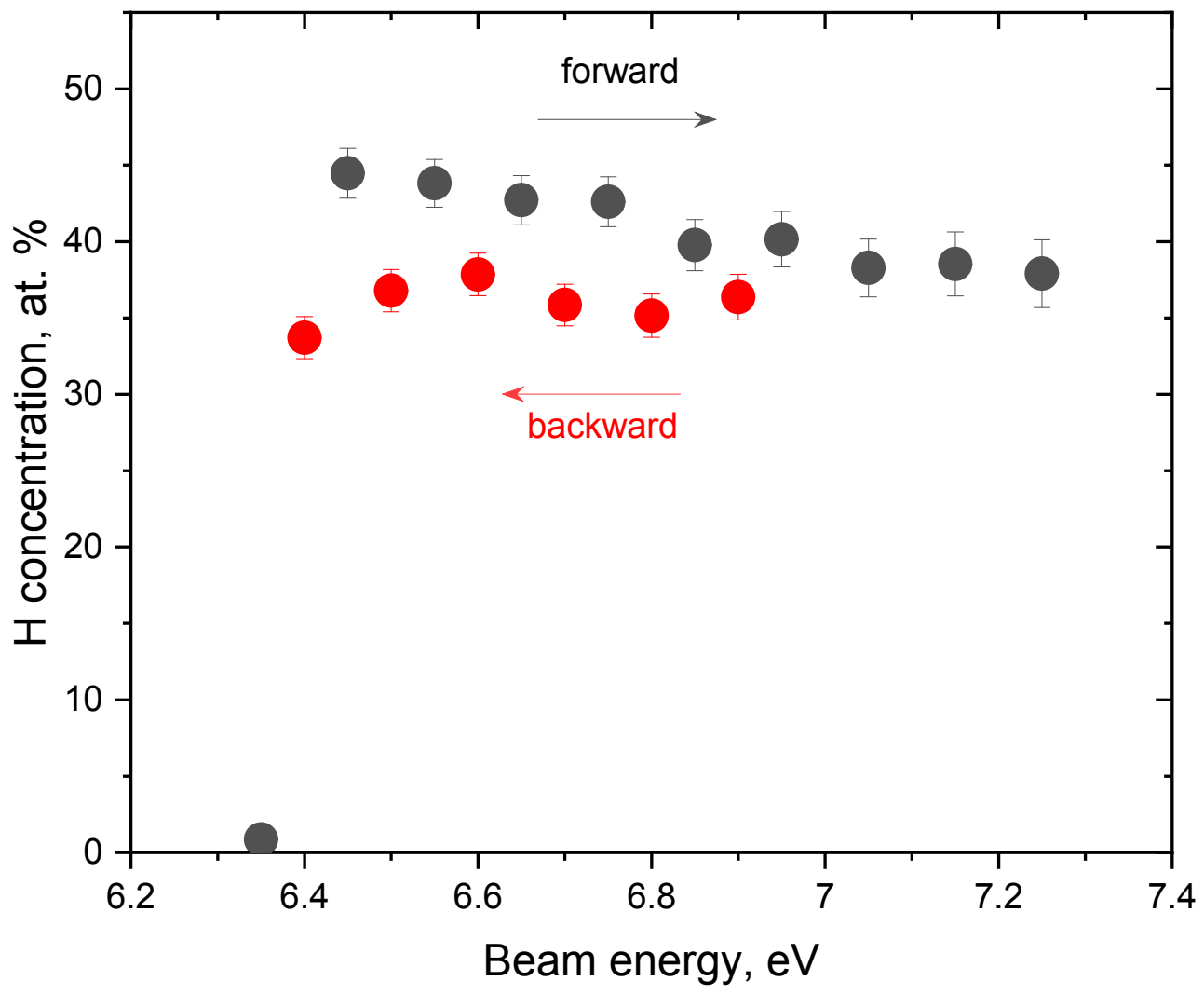

Figure S 4 Hydrogen depth profiling of benzene-derived pp:C:H NPs (30 W) using resonant NRA.

3. *N_2 adsorption/desorption isotherms*

The specific surface area and interparticle void volume were evaluated from nitrogen adsorption/desorption isotherms. Prior to the measurements, the samples (Si substrates coated with hexane-derived pp-C:H NP layers) were degassed at room temperature for 24 h. The adsorption/desorption isotherms were then recorded using high-purity nitrogen (99.999%, Linde) on an Anton Paar NOVA 3200e instrument. Each sample was measured three times, with an experimental uncertainty of approximately 10%. The Brunauer–Emmett–Teller (BET) method was used to determine the specific surface area, while pore volume was derived from the isotherms using the Barrett–Joyner–Halenda (BJH) model.

4. *VEPALS*

Positron lifetime spectra obtained in the VEPALS measurements were fitted using five lifetime components ($\tau_1 - \tau_5$) with the corresponding relative intensities ($I_1 - I_5$). The fitted parameters are shown for pp-NPs prepared from hexane in Figure S 5, from cyclohexane in Figure S 6, and from benzene in Figure S 7. The first two lifetime components (τ_1 and τ_2) lie in the sub-nanosecond range and are attributed to the annihilation of *para*-positronium (*p*-Ps) and to positrons annihilating with electrons in the atomic network of the plasma polymer matrix, respectively. The lifetime τ_3 exceeds 1 ns and is assigned to *ortho*-Positronium (*o*-Ps) annihilating in free-volume sites confined within the plasma polymer matrix. The lifetimes τ_4 and τ_5 have very low intensities, close to 1%, but were necessary to achieve satisfactory fits. The component τ_4 reaches values beyond 10 ns and is attributed to *o*-Ps annihilation in open cavities located between the pp-NPs. The lifetime τ_5 exceeds 60 ns and may indicate larger interparticle gaps or *o*-Ps escaping into vacuum. The value of τ_5 was fixed to an average value because it shows no significant trend with implantation energy and fixing it stabilizes the fitting procedure, particularly with respect to τ_3 *and* τ_4.

The positron penetration depth L presented in Figure S 5, Figure S 6, and Figure S 7, and in the main text should be regarded as a rough estimate. It was calculated for a compact (i.e., non-nanogranular) layer of ultra-high molecular polyethylene according to the formula:

$$L = A\frac{E^n}{\rho}$$

Here, E is the positron implantation energy, A = 1.813 µg cm^{-2} KeV^{-n} and n = 3.39 are empirical constants, and ρ = 0.94 g cm^3 is the density used for ultra-high molecular polyethylene.[1]

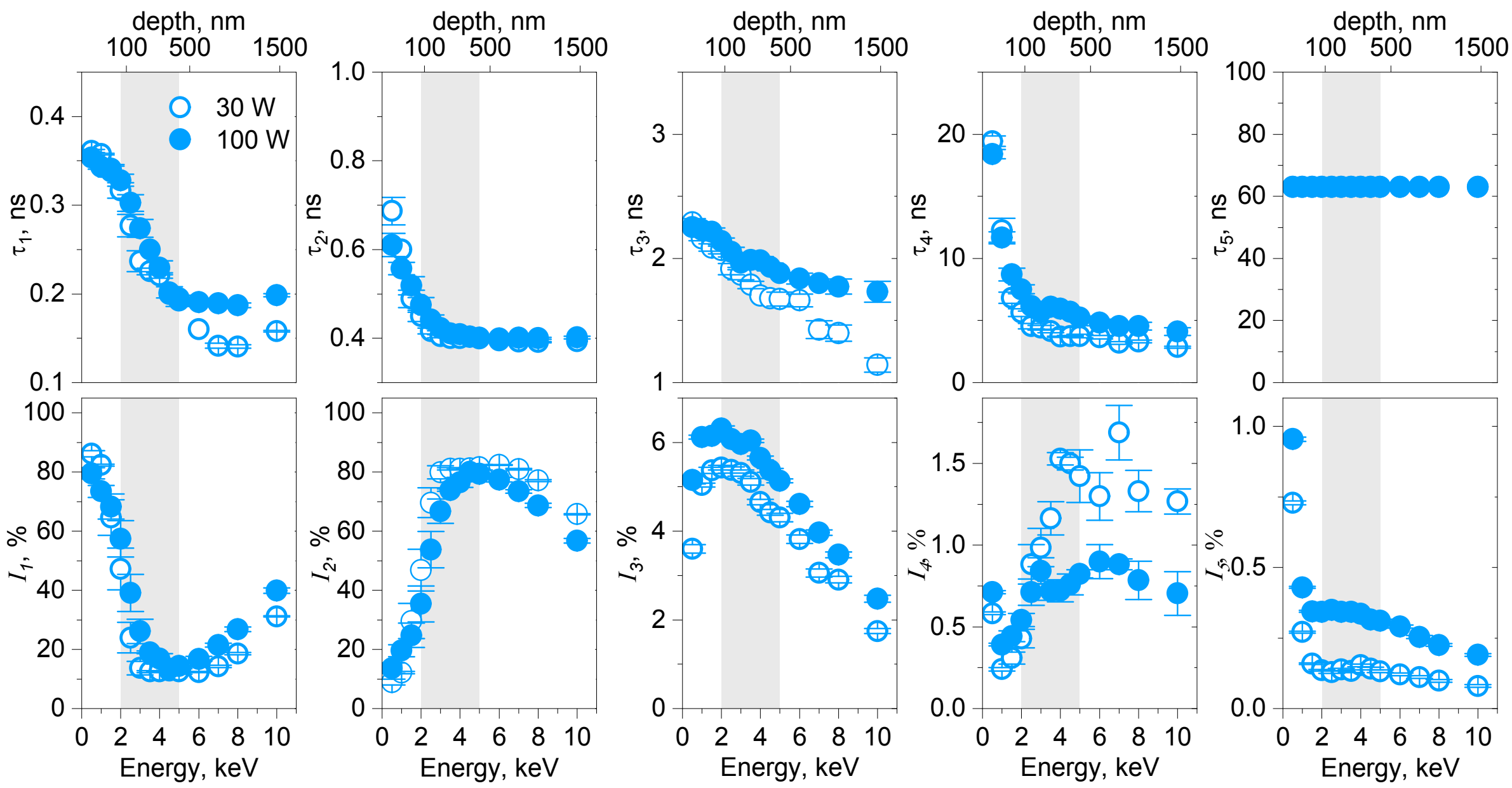

Figure S 5 Lifetimes τ and intensities I of the five components obtained from fitting the VEPALS spectra of pp-NPs prepared from hexane at 30 and 100 W. The shaded areas indicate the range of positron implantation depths corresponding to the region where the contribution from the Si substrate is minimal.

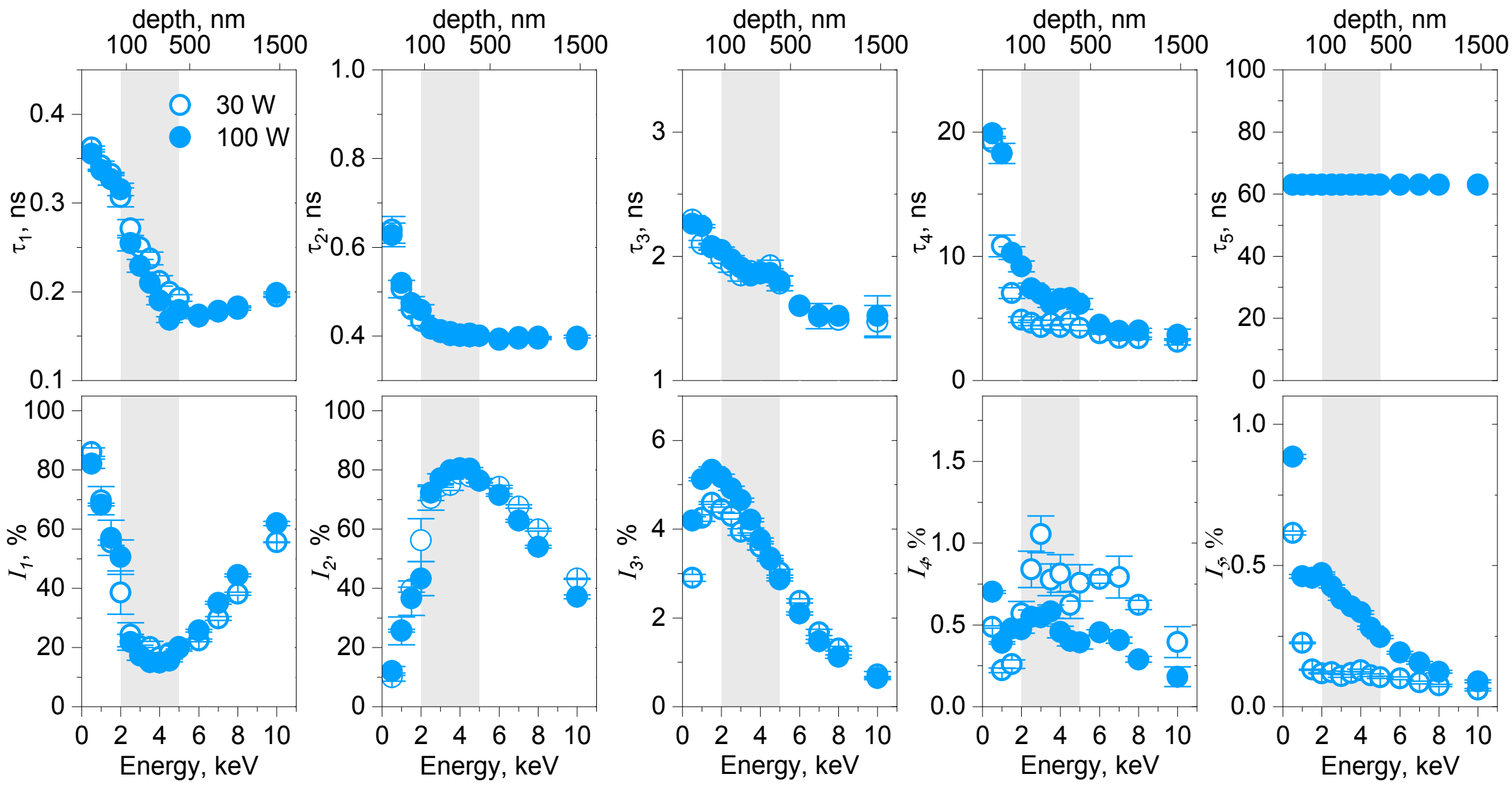

Figure S 6 Lifetimes τ and intensities I of the five components obtained from fitting the VEPALS spectra of pp-NPs prepared from cyclohexane at 30 and 100 W. The shaded areas indicate the range of positron implantation depths corresponding to the region where the contribution from the Si substrate is minimal.

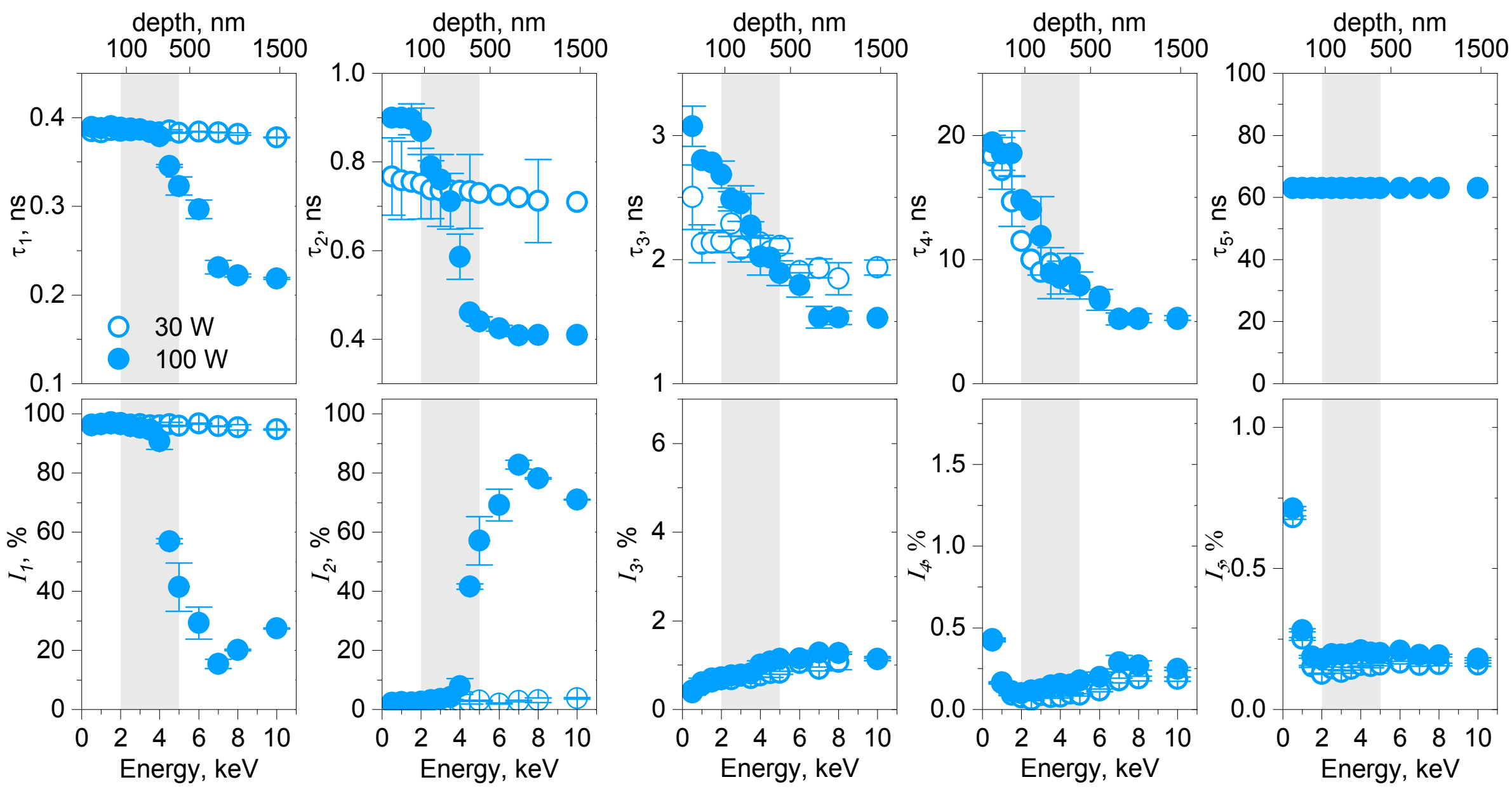

Figure S 7 Lifetimes τ and intensities I of the five components obtained from fitting the VEPALS spectra of pp-NPs prepared from benzene at 30 and 100 W. The shaded areas indicate the range of positron implantation depths corresponding to the region where the contribution from the Si substrate is minimal.

5. *Discharge power dependence of the H/C ratio and NP size*

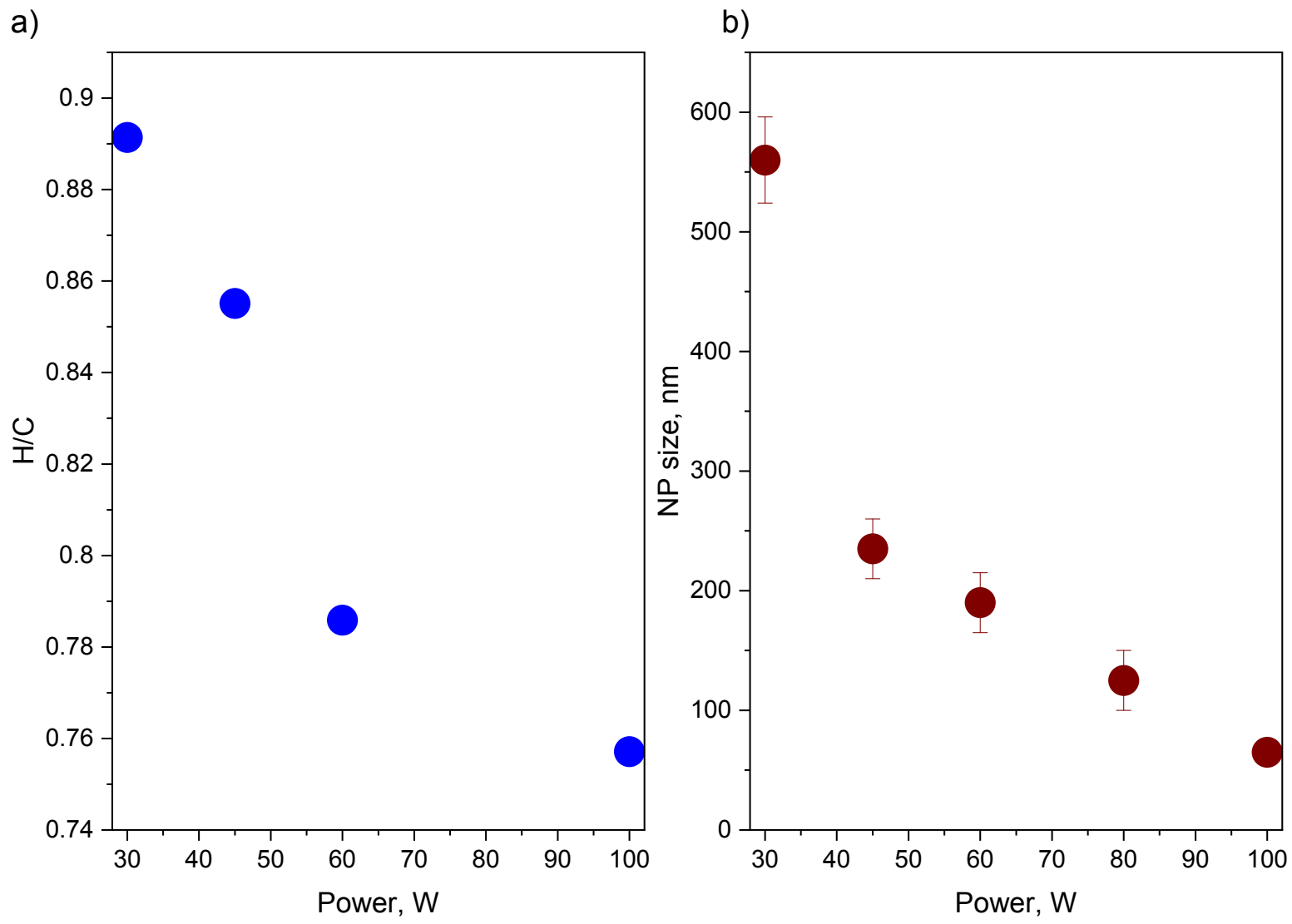

Figure S 8 Discharge power dependence of: a) H/C ratio determined by ToF-ERDA; b) NP size for hexane-derived pp-C:H NPs.

6. XPS

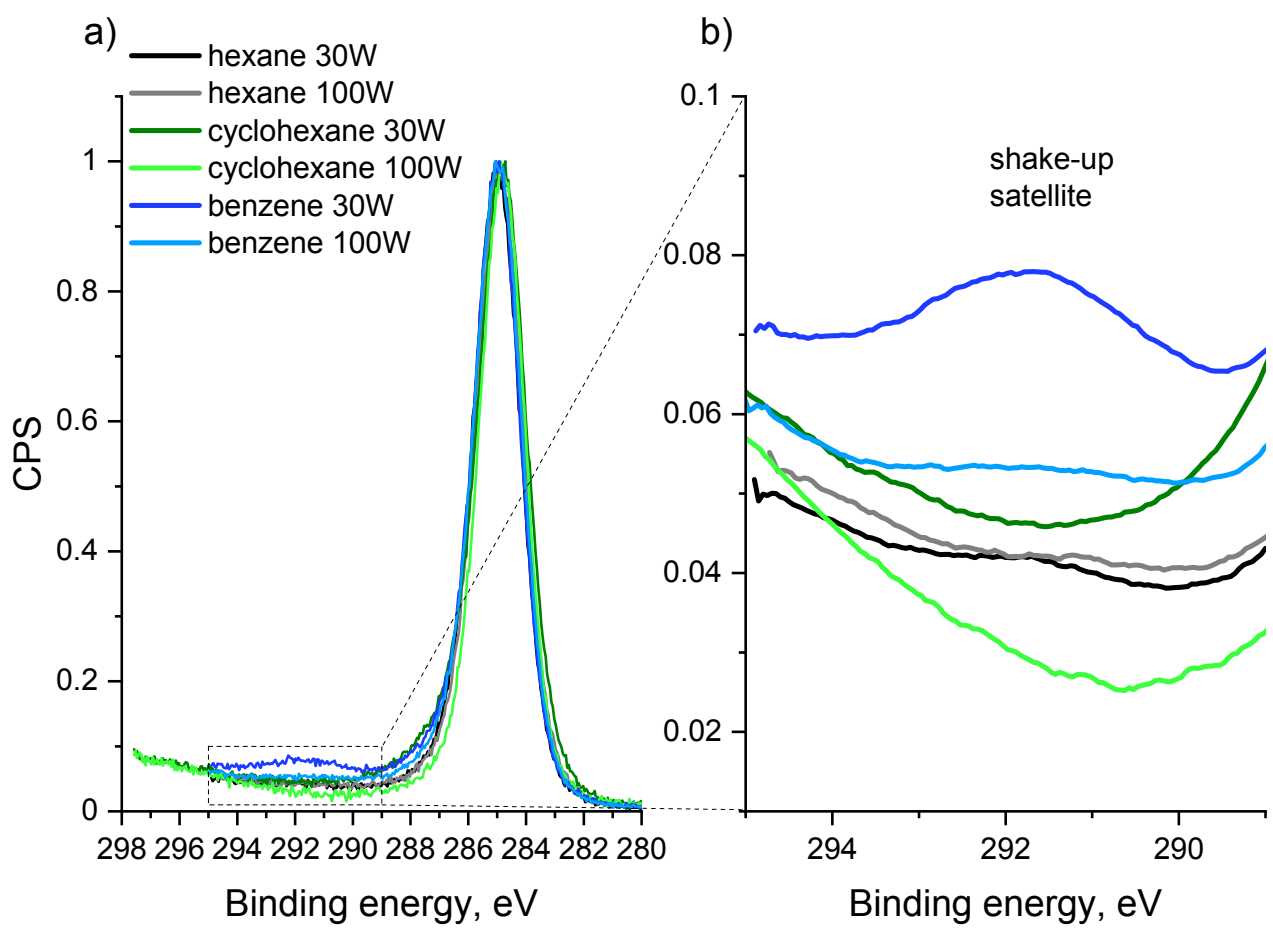

Figure S 9 C 1s XPS spectra of pp-NPs deposited from different precursors at 30 and 100 W power: a) wide binding energy range; b) magnified region showing the shake-up satellites.

7. Statistical reconstruction of plasma polymer network structure

Plasma polymerization involves a large number of simultaneous fragmentation and recombination reactions, making any mechanistic or molecular dynamics-based simulation impractical. Moreover, experimentally accessible structural information is limited to elemental composition (C and H) and qualitative FTIR and XPS signatures of a few functional groups. Oxygen is detected only in minor amounts and is most likely incorporated post deposition through reactions of atmospheric oxygen with residual radicals. Oxygen containing motifs are therefore expected to appear predominantly in terminal groups, with only negligible contribution to the carbon–carbon connectivity that determines

network topology and elasticity. Consequently, oxygen bearing species are excluded from the present TBP-based reconstruction.

7.1 Chemical Types of Units

In the present model, three chemical unit types are distinguished: carbon (C), hydrogen (H), and benzene rings. These unit types serve as the elementary constituents from which all building units (BUs) are constructed. The purpose of this coarse-grained categorization is to encode the experimentally accessible composition of pp-NPs (primarily the H/C ratio and, for some systems, the partial survival of aromatic motifs).

Benzene units are included only for pp-NPs prepared from benzene. Experimental evidence shows that a fraction of aromatic rings survives plasma polymerization of benzene, whereas pp-NPs generated from hexane or cyclohexane contain no intact benzene rings. This selectivity is therefore directly reflected in the choice of chemical unit types for individual systems.

Each unit type is associated with a formal variable Z_{type}, with type ∈ {C, H, benz} appearing in the generating functions. These variables count occurrences of the respective unit types in the reconstructed network. A benzene unit is treated as a single combinatorial entity, even though it contains six carbon atoms; this convention allows the overall carbon and hydrogen contents to be computed without introducing additional property carrying variables. Table S 1 lists the three chemical unit types and their associated symbols.

Table S 1 Chemical unit types used in the model. Benzene units are included only for pp-NPs prepared from benzene (aromatic rings partially survive plasma polymerization), whereas hexane- and cyclohexane-derived pp-NPs do not contain intact benzene rings.

№	Type label	Symbol	Variable
1	C	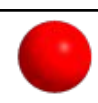	Z_{C}
2	H	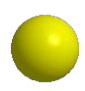	Z_{H}
3	benz	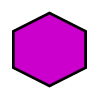	Z_{benz}

7.2 Oriented Links (OL)

Connectivity between building units is represented using five types of oriented links (OLs). Each OL corresponds to a directed chemical bond. Single, double, and triple carbon-carbon bonds are treated as symmetric OLs, meaning that their orientation does not matter. In contrast, the C–H bond is represented by an asymmetric pair of OLs: C → H and H → C.

For each OL type i, the function σ(i) identifies the corresponding OL of opposite orientation. Symmetric OLs satisfy the condition σ(i) = i, whereas the asymmetric pair (C → H, H → C) maps to each other. Table S 2 lists the five OL types together with their symbols, variables, and opposite orientations.

Table S 2 Oriented link (OL) types used to describe connectivity. For symmetric OLs (C–C single, double, triple bonds), the opposite orientation $\sigma(i)$ equals i; the asymmetric pair (C $\rightarrow$ H, H $\rightarrow$ C) maps to each other.

i	**Description**	**Symbol**	**Variable**	$\sigma(i)$
1	C–C (single)		z_1	1
2	C=C (double)		z_2	2
3	C≡C (triple)		z_3	3
4	C $\rightarrow$ H		z_4	5
5	H $\rightarrow$ C		z_5	4

7.3 Building Units (BU) and generating function (GF) of their relative molar concentrations

Building units (BUs) represent the elementary structural blocks used in the statistical reconstruction of the network. Each BU is defined by:

(i) its chemical type (C, H, benzene), and
(ii) the numbers of issued oriented links (OLs) of each type.

This results in 19 distinct BUs:

- 11 carbon-type BUs, distinguished by their numbers of OLs C–C, C=C, C≡C and C $\rightarrow$ H
- 1 hydrogen-type BU carrying a single H $\rightarrow$ C OL,
- 7 benzene-type BUs, distinguished by the numbers of OLs C–C and C $\rightarrow$ H (equivalently, by the numbers of attached C and H sites).

7.4 Parameterization of BU relative abundances

The model is fully specified by a set of five independent parameters. These parameters were chosen so that each has a direct and transparent relationship to experimentally accessible or easily estimated quantities. Although the calculation of BU concentrations in terms of these parameters is not minimal, this choice significantly simplifies the physical interpretation of the model.
To make the generating functions and intermediate expressions more intuitive, we introduce three additional dependent parameters, which will later be expressed explicitly in terms of the five independent ones.

The five independent parameters are $(c_{\mathrm{C}}, r_{\mathrm{HC}}, r_{\mathrm{benz}}, r_{21}, r_{31})$:

- c_{C}: relative molar concentration of all carbon atoms (including those in benzene rings) in the entire system (sol + gel). It is the only parameter proportional to a true molar concentration. If $c_{\mathrm{C}} = 1$, the resulting “concentration’’ of EANC corresponds to the number of EANCs per carbon atom in the system.
- r_{HC}: molar ratio $[H]/[C]$.
- r_{benz}: molar fraction of surviving benzene rings.

- r_{21}: molar ratio of double to single C–C bonds, $r_{21} = [\text{C=C}]/[\text{C–C}]$.
- r_{31}: molar ratio of triple to single C–C bonds, $r_{31} = [\text{C≡C}]/[\text{C–C}]$.
  For convenience, we introduce three additional dependent parameters:
- p_{C}: probability that a single C-issued bond connects to another carbon rather than hydrogen,
- p_2: probability that a C-type BU issues a double bond instead of two single bonds,
- p_3: probability that a C-type BU issues a triple bond.
  These probabilities will be expressed in terms of $(c_{\mathrm{C}}, r_{\mathrm{HC}}, r_{\mathrm{benz}}, r_{21}, r_{31})$ below.

7.5 Generating function (GF) of relative molar concentrations of BUs

The generating function of BU relative molar concentrations,

$$F_{\mathrm{n}}(\mathbf{Z},\mathbf{z}),\ \ \mathbf{Z} = (Z_{\mathrm{C}}, Z_{\mathrm{H}}, Z_{\mathrm{benz}}),\ \ \mathbf{z} = (z_1,\ldots,z_5),$$

is obtained directly from the definitions of the parameters:

$$F_{\mathrm{n}}(\mathbf{Z},\mathbf{z}) = c_{\mathrm{C}}$$
$$\Big((1-r_{\mathrm{benz}})\Big((1-p_3)\big((1-p_2)(p_{\mathrm{C}}z_1+(1-p_{\mathrm{C}})z_4)^2+p_2z_2\big)^2+p_3z_3(p_{\mathrm{C}}z_1+(1-p_{\mathrm{C}})z_4)\Big)Z_{\mathrm{C}}+r_{\mathrm{HC}}z_5Z_{\mathrm{H}}$$

.

Each monomial corresponds to a specific BU:

$$F_{\mathrm{n}}(\mathbf{Z},\mathbf{z}) = \sum_{\alpha=1}^{19} c_\alpha\, Z_{\mathrm{type}(\alpha)} \prod_{i=1}^{5} z_i^{n_i(\alpha)},$$

where $Z_{\mathrm{type}(\alpha)}$ denotes the variable corresponding to the chemical type of BU α, and $n_i(\alpha)$ is the number of OLs of type i issued by that BU.

All 19 BUs and their contributions to F_n are listed in Table S 3.

Table S 3 Building units (BUs) and their contributions to the number-fraction generating function $F_{\mathrm{n}}(\mathbf{Z},\mathbf{z})$ of BUs.

№	Symbol	Contribution to $F_{\mathrm{n}}(\mathbf{Z},\mathbf{z})$
1	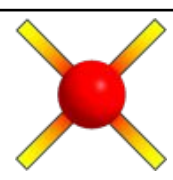	$c_{\mathrm{C}}(1-r_{\mathrm{benz}})\,(1-p_2)^2(1-p_3)(1-p_{\mathrm{C}})^4Z_{\mathrm{C}}z_4^4$
2	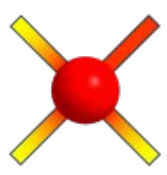	$4c_{\mathrm{C}}(1-r_{\mathrm{benz}})\,(1-p_2)^2(1-p_3)(1-p_{\mathrm{C}})^3p_{\mathrm{C}}Z_{\mathrm{C}}z_1$
3	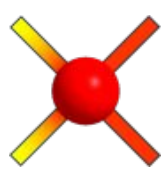	$6c_{\mathrm{C}}(1-r_{\mathrm{benz}})\,(1-p_2)^2(1-p_3)(1-p_{\mathrm{C}})^2p_{\mathrm{C}}^2Z_{\mathrm{C}}z_1^2$
4	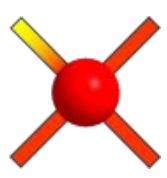	$4c_{\mathrm{C}}(1-r_{\mathrm{benz}})\,(1-p_2)^2(1-p_3)\,(1-p_{\mathrm{C}})p_{\mathrm{C}}^3Z_{\mathrm{C}}z_1^3$
5	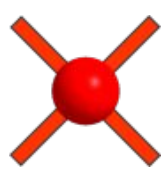	$c_{\mathrm{C}}(1-r_{\mathrm{benz}})\,(1-p_2)^2(1-p_3)p_{\mathrm{C}}^4Z_{\mathrm{C}}z_1^4$
6	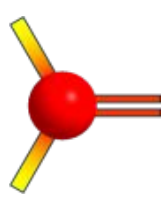	$2c_{\mathrm{C}}(1-r_{\mathrm{benz}})\,(1-p_2)p_2(1-p_3)(1-p_{\mathrm{C}})^2Z_{\mathrm{C}}z_2$
7	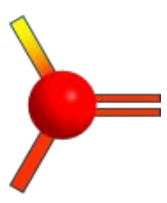	$4c_{\mathrm{C}}(1-r_{\mathrm{benz}})\,(1-p_2)p_2(1-p_3)\,(1-p_{\mathrm{C}})p_{\mathrm{C}}Z_{\mathrm{C}}z_1z_2$

8	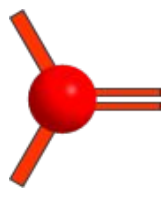	$2c_{\mathrm{C}}(1-r_{\mathrm{benz}})\,(1-p_2)p_2(1-p_3)p_{\mathrm{C}}^2Z_{\mathrm{C}}z_1^2z_2$
9	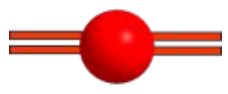	$c_{\mathrm{C}}(1-r_{\mathrm{benz}})p_2^2(1-p_3)Z_{\mathrm{C}}z_2^2$
10	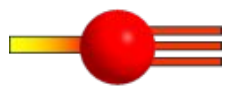	$c_{\mathrm{C}}(1-r_{\mathrm{benz}})p_3(1-p_{\mathrm{C}})Z_{\mathrm{C}}z_3z_4$
11	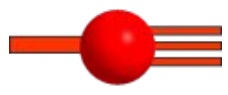	$c_{\mathrm{C}}(1-r_{\mathrm{benz}})p_3p_{\mathrm{C}}Z_{\mathrm{C}}z_1z_3$
12	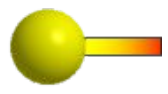	$c_{\mathrm{C}}r_{\mathrm{HC}}z_5Z_{\mathrm{H}}$
13	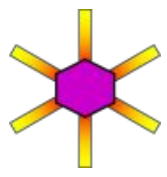	$\frac{1}{6}c_{\mathrm{C}}r_{\mathrm{benz}}(1-p_{\mathrm{C}})^6Z_{\mathrm{benz}}z_4^6$
14	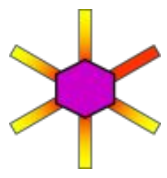	$c_{\mathrm{C}}r_{\mathrm{benz}}(1-p_{\mathrm{C}})^5p_{\mathrm{C}}Z_{\mathrm{benz}}z_1z_4^5$
15	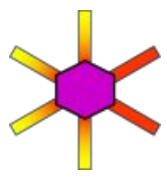	$\frac{5}{2}c_{\mathrm{C}}r_{\mathrm{benz}}(1-p_{\mathrm{C}})^4p_{\mathrm{C}}^2Z_{\mathrm{benz}}z_1^2z_4^4$
16	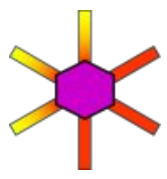	$\frac{10}{3}c_{\mathrm{C}}r_{\mathrm{benz}}(1-p_{\mathrm{C}})^3p_{\mathrm{C}}^3Z_{\mathrm{benz}}z_1^3z_4^3$
17	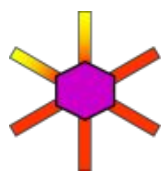	$\frac{5}{2}c_{\mathrm{C}}r_{\mathrm{benz}}(1-p_{\mathrm{C}})^2p_{\mathrm{C}}^4Z_{\mathrm{benz}}z_1^4z_4^2$
18	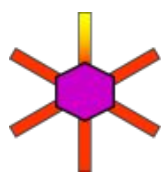	$c_{\mathrm{C}}r_{\mathrm{benz}}(1-p_{\mathrm{C}})p_{\mathrm{C}}^5Z_{\mathrm{benz}}z_1^5z_4$
19	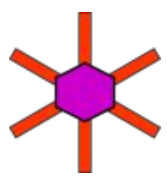	$\frac{1}{6}c_{\mathrm{C}}r_{\mathrm{benz}}p_{\mathrm{C}}^6Z_{\mathrm{benz}}z_1^6$

7.6 Branching process

Network growth is described by a branching process in which a BU is repeatedly attached via oriented links. Having selected an OL of type i, an OL of type $\sigma(i)$is randomly chosen and the corresponding BU is attached at that site.

The generating function $F_i(\mathbf{Z},\mathbf{z})$ for the probability that a BU is attached to an OL of type i (via its OL $\sigma(i)$), characterized by unit type and the numbers of OLs except the one used for attachment, is defined as:

$F_i(\mathbf{Z},\mathbf{z}) = \frac{1}{B_i}\frac{\partial F_{\mathrm{n}}(\mathbf{Z},\mathbf{z})}{\partial z_{\sigma(i)}}$, where the derivative multiplies the relative concentration of BU α by the number of OLs of type $\sigma(i)$that it issues (correctly decreasing the corresponding exponent by one).

The molar fraction of OL type i is given by:

$$B_i = \frac{\partial F_{\mathrm{n}}(\mathbf{1},\mathbf{1})}{\partial z_i}$$

with the consistency condition:

$$B_i = B_{\sigma(i)}$$

Explicit expressions for B_1,...,B_5 follow directly.

7.7 Extinction probabilities and gelation

The extinction probability v_i is the probability that a branch rooted at an OL of type i remains finite. It satisfies the self-consistency equation:

$$v_i = F_i(\mathbf{1},\mathbf{v}).$$

The trivial solution $\mathbf{v} = \mathbf{1}$ always exists. Gelation corresponds to the emergence of a nontrivial solution, detected by the singularity condition

$$\det\left(\delta_{ij} - \frac{\partial F_i(\mathbf{1},\mathbf{v})}{\partial v_j}\right) = 0.$$

7.8 Network density

An elastically active network chain (EANC) is a chain segment connecting two elastically active crosslinks (EACs). An EAC is defined as a BU that issues at least three OLs whose branches continue to infinity; its effective functionality equals the number of such infinite-continuation branches. The total number of EANCs equals half of the total number of effective infinite OLs. Effective infinite links are counted by introducing an auxiliary variable y, distinguishing finite and infinite continuations:

$$n_\mathrm{e} = \frac{1}{2}\left[\frac{\partial}{\partial y}F_\mathrm{n}(\mathbf{1},\mathbf{v}+(\mathbf{1}-\mathbf{v})y)\Big|_{y\to1} - \frac{\partial}{\partial y}F_\mathrm{n}(\mathbf{1},\mathbf{v}+(\mathbf{1}-\mathbf{v})y)\Big|_{y\to0} - \frac{\partial^2}{\partial y^2}F_\mathrm{n}(\mathbf{1},\mathbf{v}+(\mathbf{1}-\mathbf{v})y)\Big|_{y\to0}\right].$$

The interpretation of n_e depends on the normalization of carbon concentration c_C. With the convention $c_\mathrm{C} = 1$ for pp-NPs comprising sol+gel, n_e gives the number of EANCs per carbon atom (including carbons in benzene rings).

7.9 Reparametrization

The probabilities p_C,p_2,p_3 are expressed in terms of the directly accessible ratios

$$(r_\mathrm{HC},r_\mathrm{benz},r_{21},r_{31})$$

through the definitions

$$r_{21} = \frac{B_2}{B_1}, r_{31} = \frac{B_3}{B_1}, B_4 = B_5.$$

Solving these relations yields:

$$\mathrm{p_C} = \frac{(1-r_\mathrm{HC}) + 3(1-r_\mathrm{benz})}{(1-r_\mathrm{HC}) + 3(1-r_\mathrm{benz}) + (1+2r_{21}+3r_{31})r_\mathrm{HC}}$$

$$p_2 = \frac{r_{21}}{2} \frac{(1 - r_{\mathrm{HC}}) + 3(1 - r_{\mathrm{benz}})}{(1 + 2r_{21} + 3r_{31})(1 - r_{\mathrm{benz}}) - r_{31}\big((1 - r_{\mathrm{HC}}) + 3(1 - r_{\mathrm{benz}})\big)}$$

$$p_3 = r_{31} \frac{(1 - r_{\mathrm{HC}}) + 3(1 - r_{\mathrm{benz}})}{(1 + 2r_{21} + 3r_{31})(1 - r_{\mathrm{benz}})}$$

7.10 Two extreme cases of pp NPs and calculation of input parameters

We distinguish two limiting scenarios:

- sol + gel case: pp-NPs include both sol molecules and gel,
- gel-only case: pp-NPs comprise only the gel fraction.

In the main text, pp-NPs are treated as sol+gel. In practice, however, low-molecular sol species (e.g., methane, ethane) may escape the particles. Hydrogen molecules are not included because H–H OLs and corresponding BUs are not part of the model. Actual pp-NPs are expected to lie between these two extremes.

Even though the absolute value of network density is lower in the gel-only case, the trends across systems remain the same, and therefore only the sol+gel case is discussed in the main text.

In the sol+gel case, input parameters are assigned directly from experiment:

$$c_{\mathrm{C}} = 1,\ r_{\mathrm{HC}} = r_{\mathrm{HC}}^{\mathrm{exp}},\ r_{\mathrm{benz}} = r_{\mathrm{benz}}^{\mathrm{est}},\ r_{21} = r_{21}^{\mathrm{est}},\ r_{31} = r_{31}^{\mathrm{est}}.$$

In the gel-only case, the parameters are obtained from six relative molar concentrations (for carbon, hydrogen, and benzene BUs, and for the OLs C–C, C=C, C≡C). These six quantities correspond to derivatives of $F_n(\mathbf{Z},\mathbf{z})$ with respect to

$$Z_{\mathrm{C}}, Z_{\mathrm{H}}, Z_{\mathrm{benz}}, z_1, z_2, z_3$$

evaluated at $(\mathbf{Z},\mathbf{z}) = (\mathbf{1},\mathbf{1})$ for the entire system (sol+gel) and $(\mathbf{Z},\mathbf{z}) = (\mathbf{1},\mathbf{v})$ for sol only. The gel contribution is the difference between these values.

Let

$$d_i \ \ (\mathrm{i} = 1,\ldots,6)$$

denote the six quantities for sol+gel,

$d_{i,\mathrm{s}}$ for sol, and

$d_{\mathrm{i,g}} = d_i - d_{i,\mathrm{s}}$ for gel.

The five gel-fraction parameters satisfy:

$$d_{1,\mathrm{g}} + 6d_{3,\mathrm{g}} = 1,\ \frac{d_{2,\mathrm{g}}}{d_{1,\mathrm{g}} + 6d_{3,\mathrm{g}}} = r_{\mathrm{HC}}^{\mathrm{exp}},\ \frac{6d_{3,\mathrm{g}}}{d_{1,\mathrm{g}} + 6d_{3,\mathrm{g}}} = r_{\mathrm{benz}}^{\mathrm{est}},$$

$$\frac{d_{5,\mathrm{g}}}{d_{4,\mathrm{g}}} = r_{21}^{\mathrm{est}},\ \frac{d_{6,\mathrm{g}}}{d_{4,\mathrm{g}}} = r_{31}^{\mathrm{est}}$$

Solving this system yields

$(c_{\mathrm{C}}, r_{\mathrm{HC}}, r_{\mathrm{benz}}, r_{21}, r_{31})$ for the gel-only case.

Note that computing $d_{i,\mathrm{s}}$ requires solving the extinction-probability equations. The corresponding identities for sol+gel (with d_i instead of $d_{i,\mathrm{g}}$) yield directly the five input parameters, confirming consistency.

7.11 Experimental and estimated input parameters and network densities

The values of

$$(c_{\mathrm{C}}, r_{\mathrm{HC}}, r_{\mathrm{benz}}, r_{21}, r_{31})$$

and the corresponding network densities n_e for the six systems (hexane30, hexane100, cyclohexane30, cyclohexane100, benzene30, benzene100) are summarized in Table S 4.

Table S 4 Values of the five input parameters (c_C,r_{HC},r_{benz},r_{21},r_{31}) for the six investigated systems (hexane30, hexane100, cyclohexane30, cyclohexane100, benzene30, benzene100) in the sol+gel and gel-only scenarios, together with the corresponding network densities n_e.

		hexane30	hexane100	cyclohexane30	cyclohexane100	benzene30	benzene100
c_C	sol + gel	1	1	1	1	1	1
	gel only	1.0370	1.1203	1.0341	1.0785	1.0023	1.0032
r_{HC}	sol + gel	0.83	0.78	0.81	0.69	0.49	0.46
	gel only	0.8566	0.8304	0.8346	0.7261	0.4924	0.4629
r_{benz}	sol + gel	0	0	0	0	0.05	0.04
	gel only	0	0	0	0	0.0499	0.0399
r_{21}	sol + gel	0.1875	0.2857	0.1875	0.2857	0.0652	0.1023
	gel only	0.201	0.3103	0.2003	0.3033	0.0664	0.1040
r_{31}	sol + gel	0.0625	0.1429	0.0625	0.1429	0.0217	0.0341
	gel only	0.091	0.2292	0.0900	0.2141	0.0256	0.0402
n_e	sol + gel	0.7278	0.4648	0.7448	0.5311	1.3263	1.2357
	gel only	0.6279	0.3073	0.6482	0.3932	1.3054	1.2067

The absolute values of the network density differ between the sol+gel and gel-only cases; however, the trends across systems are identical. Accordingly, only the sol + gel values are discussed in the main text.

Contrary to intuitive expectations, the gel-only case yields a lower network density. The same experimental inputs (H/C ratio and the ratios of double and triple to single C–C bonds) imply similar average crosslink functionality when applied to the entire system in the sol + gel case and to the gel fraction in the gel-only case. Because the sol fraction contains units of lower functionality, inclusion of the sol reduces the overall average functionality of the system. Since TBP is defined with respect to the whole system, this reduction leads directly to a lower network density, n_e, in the gel-only case.

The crosslink density decreases with increasing r_{HC}, r_{21}, and r_{31}, as hydrogen atoms and multiple C–C bonds reduce crosslink functionality. Consistent with this interpretation, these parameters are higher in the gel-only case than in the sol + gel case. By contrast, r_{benz}exhibits only weak, system-dependent variation and remains approximately constant. The carbon abundance, c_{C}, is necessarily higher in the gel-only case because the gel fraction is normalized to unity, whereas the sol fraction contributes additional carbon atoms in the sol + gel representation.

7.12 Additional graphical illustration of TBP model behavior

To complement the parameter tables and to document the qualitative behavior of the TBP-based reconstruction, several auxiliary plots are provided below. These plots are not required for the main discussion, as the network densities listed in Table S 4 already capture all system-specific trends. Their purpose is solely to illustrate that the model responds smoothly and monotonically to changes in the underlying compositional parameters, without nonlinearities or unexpected features.

A 3D contour plot of the network density n_e as a function of the three compositional parameters (r_{HC},r_{benz},r_{21}) is shown in the main text. This figure represents the global behavior of the model, since all three quantities vary freely.

The 2D contour plots presented below represent two-parameter sections through the same three-dimensional parameter space. In all contour plots we set $c_{\mathrm{C}} = 1$, so that the network density n_e is expressed per carbon atom in the reconstructed system, and we set $r_{31} = 0$ because only a qualitative indication of triple-bond content is available and its estimated values for the benzene-derived systems are small and system-dependent; fixing $r_{31} = 0$ simplifies the graphical representation without affecting the qualitative trends.

In each contour plot, two of the quantities (r_{HC},r_{benz},r_{21}) vary freely, while the third is fixed to the value corresponding to either the benzene30 or the benzene100 system. In this context, the labels “benzene30’’ and “benzene100’’ refer only to the fixed value of that single parameter; the remaining parameters vary freely. Thus, the contour plots do not represent physical samples but rather illustrate the behavior of the model in the vicinity of two relevant reference points.

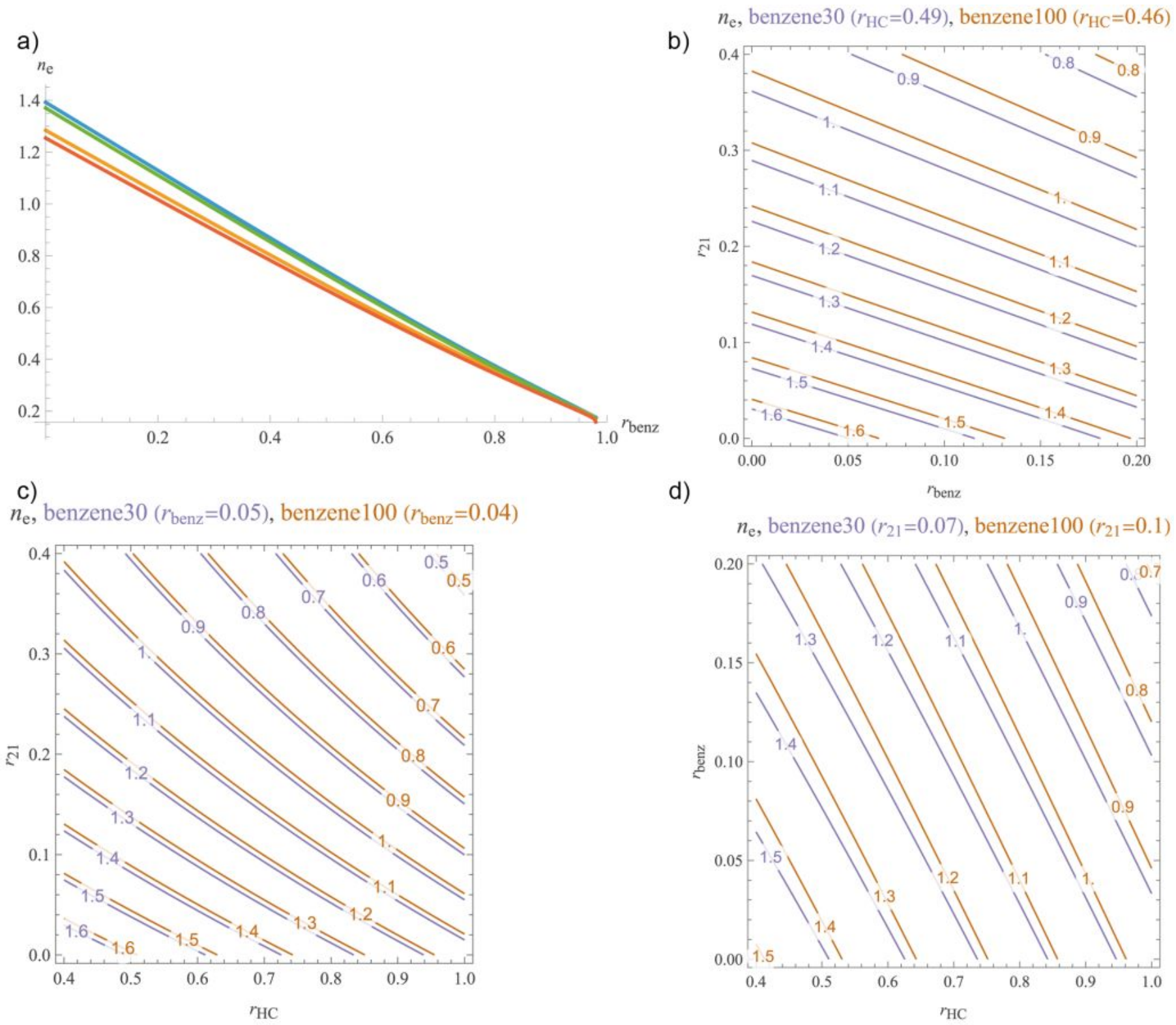

Figure S 10 Additional graphical illustration of the behavior of the TBP reconstruction: a) dependence of n_{e} on r_{benz} for the benzene30 and benzene100 reference sets, shown for both the sol+gel and gel-only reconstructions. In each curve, the remaining four parameters (c_{C},r_{HC},r_{21},r_{31}) are set to the values of the corresponding system and scenario. All four curves are nearly linear and monotonic. (b–d) Contour plots of n_{e} for selected pairs of parameters among (r_{HC},r_{benz},r_{21}), with the third parameter fixed as described above and with $c_{\mathrm{C}} = 1$and $r_{31} = 0$. The contours are smooth, nearly linear and monotonic.

These plots collectively demonstrate that the TBP reconstruction behaves stably throughout the relevant parameter space: small changes in the compositional quantities lead to gradual, almost linear changes in the predicted network density.

References

(1) Dryzek, J.; Horodek, P. GEANT4 Simulation of Slow Positron Beam Implantation Profiles. *Nucl. Instruments Methods Phys. Res. Sect. B Beam Interact. with Mater. Atoms* **2008**, *266* (18), 4000–4009. https://doi.org/10.1016/j.nimb.2008.06.033.
